# Insecticidal Activities and GC-MS Analysis of the Selected Family Members of Meliaceae Used Traditionally as Insecticides

**DOI:** 10.3390/plants11223046

**Published:** 2022-11-10

**Authors:** Kolwane Calphonia Shilaluke, Annah Ntsamaeeng Moteetee

**Affiliations:** Department of Botany and Plant Biotechnology, University of Johannesburg, P.O. Box 524, Auckland Park 2006, South Africa

**Keywords:** antifeedants, botanical insecticides, insect pests, Meliaceae, synthetic pesticides

## Abstract

The environmental and health risks associated with synthetic pesticides have increased the demand for botanical insecticides as safer and biodegradable alternatives to control insect pests in agriculture. Hence in this study, five Meliaceae species were evaluated for their insecticidal activities against the *Spodoptera frugiperda* and the *Plutella xylostella* larvae, as well as their chemical constituents. Repellence, feeding deterrence, and topical application bioassays were employed to evaluate their insecticidal activities. GC-MS analysis was performed to identify chemical compounds present in each plant. The repellence bioassay indicated that *Melia azedarach* extracts exhibited the highest repellence percentage against *S. frugiperda* (95%) and *P. xylostella* (90%). The feeding deterrence bioassay showed that *M. azedarach* and *Trichilia dregeana* extracts displayed excellent antifeeding activity against the *S. frugiperda* (deterrent coefficient, 83.95) and *P. xylostella* (deterrent coefficient, 112.25), respectively. The topical application bioassay demonstrated that *Ekebergia capensis* extracts had the highest larval mortality against *S. frugiperda* (LD_50_ 0.14 mg/kg). Conversely, *M. azedarach* extracts showed the highest larval mortality against *P. xylostella* (LD_50_ 0.14 mg/kg). GC-MS analysis revealed that all plant extracts had compounds belonging to the two noteworthy groups (phenols and terpenes), which possess insecticidal properties. Overall, this study lends scientific credence to the folkloric use of Meliaceae species as potential biocontrol agents against insect pests.

## 1. Introduction

The agricultural sector has always been faced with challenges due to insect pests and will continue to do so in the future [1]. These pests damage crops during the growing period, and they may also subsequently cause damage to the harvested products stored in storehouses [2]. Controlling insect pests remains a problem as the insects keep building resistance to common pesticides while, on the other hand, toxic pesticides are being removed from the markets [1]. Synthetic pesticides have been commonly used and are considered a highly effective means of controlling plant damage caused by insects [3], which leads to remarkable improvements in plant yield productivity [4]. However, the indiscriminate and haphazard usage of synthetic pesticides has adversely affected human health and the ecosystem as a whole [5].

The presence of pesticide residues in foods, fruits, vegetables, and even in breast-feeding mothers’ milk creates a threat to human health. In developing countries, nearly 3 million farmworkers experience severe pesticide poisoning, resulting in about 18,000 deaths, while 25 million workers suffer from mild pesticide poisoning each year [6]. The use of synthetic pesticides also raises several environmental concerns because over 5% of the sprayed synthetic pesticides do not reach their target insect pests; instead, they can be found in air, soil, and water streams [7]. As a result of these devastating occupational synthetic pesticide poisoning cases, research to find alternative methods that are environmentally friendly and cost-effective in controlling insect pests has increased [1].

Based on recent studies to find ways to mitigate problems caused by synthetic pesticides, natural bio-insecticides from medicinal plants can be an excellent alternative strategy to overcome pest resistance and environmental contamination [8]. This possibility is not surprising as plants are rich sources of bioactive chemicals, and botanical insecticides have been reported to have fewer adverse effects on the environment or human health [1].

Meliaceae is one of two flowering plant families that have gained considerable attention, whereby systematic investigations of its members for their insecticidal potential have been undertaken [9,10]. Chemicals extracted from members of the Meliaceae have received attention recently from applied entomologists due to their excellent properties as control agents for insects [11]. This knowledge has prompted the interest to assess other family members for their insecticidal and antifeedant properties in this study.

*Plutella xylostella* (L.) (Lepidoptera: Plutellidae), commonly known as the diamondback moth (DBM) or the cabbage moth, is an economically important pest of cruciferous plants globally [12]. The Diamondback moth is an oligophagous insect that mainly feeds on cole crops, including broccoli, brussels sprouts, canola, cauliflower, and cabbage, which are of essential economic value [13]. The insect is important in agriculture as causes yield losses of as much as 100% [14]. In the 1970s, there was a major outbreak of DBM, mainly due to the development of resistance to synthetic insecticides [13]. It has been estimated that the yield losses and control associated with diamondback moth globally ranged between 4–5 US billion dollars yearly [14]. In sub-Saharan Africa, crop losses due to diamondback moths have been reported to be between 8–22% in the field [15].

*Spodoptera frugiperda* (J.E. Smith) (Lepidoptera: Noctuidae), commonly known as the fall armyworm (FAW), is a polyphagous insect that is important in agriculture as it is difficult to control and, as a result, causes a lot of damage [16]. This migratory insect also causes enormous economic losses, mainly attacking crops that form part of the primary staple food [17], including rice, maize, forage grasses, sorghum, alfalfa, vegetable crops, and many others [16]. The first case to be reported in Africa of the fall armyworm was in late 2016, when it attacked most West African farms and subsequently spread throughout the continent rapidly and is now found in 44 African countries [18]. Environmentally friendly and effective methods to control fall armyworms are crucial as these insects are heavy foliage feeders [17] and can result in the total loss of crops. In sub-Saharan Africa, maize, rice, sorghum, and sugarcane crop damage is estimated to cause up to USD 13 billion yearly [19].

Ever since plant-derived products have gained increased attention from researchers to assess their insecticidal properties, more than 2000 plant species have been recorded to be used traditionally as insecticides [20]. However, many studies that attempted to validate these properties scientifically are incomplete; the bioassays procedures used were usually inappropriate or inadequate [21]. As a result, biological compounds that are potentially useful remain uninvestigated, undiscovered, underutilized, or undeveloped from this reservoir of unstudied plant materials [22]. Hence, in this study, four Meliaceae species that had previously not been evaluated extensively for their insecticidal and antifeedant properties against the test insects *S. frugiperda* and *P. xylostella* were selected. *Melia azedarach* was chosen as a positive control as it is a well-known bioinsecticide plant. Water extracts were selected for extraction in this study because it is one of the simplest and safest (non-toxicity) solvents. In addition, aqueous plant extracts are traditionally used to control insect pests. Using aqueous extracts is fitting because the main purpose of this study is to identify safer, cost-effective, and renewable alternative methods to synthetic pesticides. Slightly polar acetone and ethanol extracts were selected because the main targeted compounds, limonoids (terpenes), were reported to have a higher solubility in polar solvents and alcohol [23].

## 2. Results

### 2.1. Antifeedant and Insecticidal Analysis

#### 2.1.1. Repellence Test

##### Repellence Bioassay against *S. frugiperda* Larvae

Table 1 indicates the results of the repellence bioassay test of the five selected Meliaceae species against the *S. frugiperda*. Positive average percentage repulsion values exhibit repellence, and negative average percentage repulsion values exhibit attractancy. Plant extracts that are ranked in higher classes (i.e., III, IV, and V) are considered to have a high repellence against the larvae, and those that are ranked to lower classes (I and II) have partial repellence against the larvae. Aqueous and ethanolic extracts of *Melia azedarach* L. and *Trichilia dregeana* Harv. & Sond. acetone extracts were found to have strongly repelled the *S. frugiperda* larvae with repellence of 95%, 65%, and 71%, and they belonged to class V, IV, and IV, respectively. It is followed by aqueous extracts of *Turraea floribunda* Hochst. (49%) belonging to class III. Aqueous and ethanol extracts of *T. dregeana* and ethanolic extracts of *Turraea obtusifolia* Hochst. moderately repelled the *S. frugiperda* with repellence of 40%, 30%, and 30%, respectively. Aqueous and acetone extracts of *T. obtusifolia* were recorded to have the lowest repellency (3% and 5%) and were all assigned to the lowest class (I). Ethanolic extracts of *T. floribunda* (−55%) indicated *S. frugiperda* larvae stimulation.

##### Repellence Bioassay against *P. xylostella* Larvae

Table 2 indicates the average percentage repulsion for the five Meliaceae species screened for their repellence activity against *P. xylostella* larvae. The overall highest percentage repulsion against the *P. xylostella* larvae was recorded for the aqueous (90%) and ethanol (80%) extracts of *Melia azedarach*, meaning that they exhibited excellent repellent activity, hence they were assigned to classes V and IV, respectively. Good repellent activity against *P. xylostella* was also recorded for acetone (65%) and ethanol (65%) extracts of *T. dregeana*, and they were assigned to class IV. Extracts of *E. capensis* moderately repelled *P. xylostella* larvae with repellence of 60% (aqueous), 50% (acetone), and 50% (ethanol), assigned to class III. All extracts of *T. obtusifolia*, i.e., aqueous (15%), acetone (30%), and ethanol (31%), recorded the lowest repellent activities against *P. xylostella.* Ethanolic extracts of *T. floribunda* (−10%) indicated the *P. xylostella* larvae stimulation.

#### 2.1.2. Feeding Deterrence Test

##### Feeding Deterrence Activity of *S. frugiperda* Larvae

Table 3 indicates the feeding deterrent activity coefficients of Meliaceae species against the fall armyworm larvae. All extracts exhibited feeding activity against the larvae to a certain extent, except for the ethanolic extracts of *T. floribunda*, which were found to have inert antifeedant compounds against the *S. frugiperda* larvae with a feeding deterrent coefficient of −12.89. Of all the tested extracts, aqueous extracts of *M. azedarach* (83.92) and aqueous (68.44) and ethanol (67.29) extracts *T. obtusifolia* recorded the highest coefficient of deterrence, indicating a good feeding deterrence activity. Aqueous extracts of *T. floribunda* and aqueous extracts of *T. dregeana* moderately caused larvae fertility, with feeding deterrence coefficients of 66.96 and 62.02, respectively, ranked ++. Furthermore, ethanolic extracts of *E. capensis* and acetone extracts of *T. floribunda* were the least effective feeding deterrents against the *S. frugiperda* larvae.

##### Feeding Deterrence Activity of *P. xylostella* Larvae

Table 4 indicates the feeding deterrent activities of the studied Meliaceae species against the diamondback moth larvae. All plant extracts exhibited noteworthy deterrence against the *P. xylostella* larvae. All extracts of *T. dregeana* showed exceptionally high feeding deterrent activities, with acetone recording a 112.25 deterrence coefficient ranked +++, ethanol (99.39, ++), and aqueous (98.77, ++). Aqueous extracts of *T. obtusifolia* and ethanolic extracts of *E. capensis* moderately caused feeding deterrence of the larvae, with feeding coefficients of 86.74 and 85.79, respectively, both ranked ++. Meanwhile, aqueous (13.95, +) and acetone (25.93, +) extracts of *T. floribunda* were the least effective feeding deterrents against *P. xylostella* larvae.

#### 2.1.3. Topical Application Test

##### Contact Toxicity against *S. frugiperda* Larvae

Table 5 shows the direct contact toxicity of the Meliaceae plant extracts to the *S. frugiperda* larvae using different concentrations. Aqueous extracts of *T. dregeana* and *M. azedarach* exhibited a positive correlation, where the least concentrated extracts [0.5] showed less toxicity than the more concentrated extracts [1.0]. At [0.5], extracts recorded a 20% mortality rate, while [1.0] recorded an 80% mortality rate. The negative correlation between the concentration of extracts and the rate of mortality observed was recorded for *E. capensis* (acetone), *M. azedarach* (acetone and ethanol), and *T. floribunda* (acetone), where at [0.5] 20%, the mortality rate and at [1.0] mortality rate was 80%. Extracts that did not show any correlation and had constant mortality rates were aqueous extracts of *E. capensis* where, at [0.5] and [1.0], 80% of the larvae died, aqueous extracts of *T. floribunda* at [0.5] and [1.0] caused 60% mortality, and ethanolic extracts of *T. floribunda* at [0.5] and [1.0] caused 20% larval mortality. Probability unit (Probit) analysis showed that aqueous extracts of *E. capensis* (LD_50_ value of 0.14 mg/kg) and *T. floribunda* (LD_50_ value of 0.56 mg/kg) were more toxic to the *S. frugiperda* larvae. Probit analysis also indicated that ethanolic extracts of *E. capensis* were the least toxic to the fall armyworm, with LD_50_ values of 851.14 mg/kg.

##### Contact Toxicity against *P. xylostella* Larvae

Results of the direct contact toxicity of the Meliaceae plant extracts to the *P. xylostella* larvae using different concentrations are outlined in Table 6. All extracts of *M. azedarach* at 500 ppm and 1000 ppm concentrations showed excellent results, as they killed 80% of the *P. xylostella* larvae. The Probit analysis further supported this and indicated that all three different extracts of *M. azedarach* were the most toxic to *P. xylostella*, with LD_50_ of 0.14 mg/kg. Results for acetone extracts of *E. capensis* and ethanolic extracts of *T. floribunda* showed a positive correlation between the concentration of extracts and mortality rates recorded. At [0.5], *E. capensis* and *T. floribunda* recorded a mortality of 20%, and at [1.0], they recorded an 80% mortality rate. The negative correlation between the concentration of extracts and the rate of mortality observed was recorded for acetone extracts of *T. dregeana* and aqueous extracts of *T. floribunda*. At [0.5], both extracts killed 80% of the larvae; at [1.0], *T. dregeana* killed 20%, while *T. floribunda* killed 40% of the larvae. Extracts that did not show any correlation and had a constant mortality rate were aqueous extracts of *T. obtusifolia* because, at [0.5] and [1.0], the extracts killed 40% of the *P. xylostella* larvae. Probit analysis indicated that only the aqueous extract of *T. obtusifolia* was the second most toxic to the *P. xylostella* larvae, with an LD_50_ value of 1.78 mg/kg. Meanwhile, all other plant extracts displayed insignificant toxicity to the *P. xylostella*, with acetone and ethanol extracts of *T. obtusifolia* recording the highest LD_50_ value of 1318.26 mg/kg.

### 2.2. GC-HRT-MS Analyses

The presence of chemical compounds in plants is important as they may be responsible for their biological activities, antifeedant and insecticidal properties. Table 7, Table 8, Table 9, Table 10, Table 11, Table 12, Table 13 and Table 14 indicate active compounds present in each Meliaceae species using GC-MS analyses, with their retention time (RT), observed mass to charge ion ratio (*m/z*), molecular formula (MF), metabolite class (MC), and fold change (FC, the average of the peak area values obtained at the different injections of the same compound). In *E. capensis* acetone extracts, thirty-three compounds were identified (Table 7), most of which are triterpenoids (five), alkanes (three), esters (three), sesquiterpenoids (three), diterpenoids (two), methyl esters (two), and two compounds were unclassified. Ethanolic extracts of *E. capensis* in Table 8 identified fifty compounds, of which most are sesquiterpenoids (eight), fatty acids (five), diterpenoids (three), methyl esters (three), triterpenoids (three), benzofurans (two), esters (two), fatty amides (two), and one compound was unclassified.

Table 9 shows the results of acetone extracts of *M. azedarach*; twenty-six compounds were identified. Of these, two are classified as esters, alkanes (three), diterpenoids (two), methyl esters (two), non-metal compounds (two), organosilicons (two), and triterpenoid (two). Thirty-three compounds were identified in ethanolic extracts of *M. azedarach*, shown in Table 10. Four compounds are classified as esters, diterpenoids (three), fatty acids (three), fatty amides (three), hydrocarbons (two), ketones (two), steroids (two), triterpenoids (two), and three compounds were unclassified.

Thirty-nine compounds were identified in acetone extracts of *T. dregeana* (Table 11), most of which are diterpenoids (six), esters (four), triterpenoids (four), alkanes (three), fatty acids (two), non-metal compounds (two), steroids (two), and one compound was unclassified. Forty-three compounds were identified in ethanolic extracts of *T. dregeana* (Table 12), with most of the compounds in the classes: diterpenoids (seven), sesquiterpenoids (five), fatty acids (four), triterpenoids (four), steroids (three), alcohols (two), esters (two), fatty amides (two), and one compound was unclassified.

Acetone leaf extracts of *T. floribunda* (Table 13) had sixty compounds, of which seven are diterpenoids (seven), sesquiterpenoids (seven), alkanes (four), methyl esters (four), triterpenoids (four), non-metal compounds (three), esters (three), fatty acids (two), fatty alcohols (two), hydrocarbons (two), and three compounds were unclassified. Table 14 shows thirty compounds identified in ethanol extracts of *T. floribunda*; most of the chemical compounds belong to the chemical classes: fatty acids (four), diterpenoids (three), esters (two), methyl esters (two), non-metal compounds (two), and one compound was unclassified.

Table 15 shows forty-four compounds identified in acetone extracts of *T. obtusifolia*; chemical classes with the most chemical compounds are: fatty acids (six), sesquiterpenoids (five), alkanes (three), diterpenoids (three), triterpenoids (three), esters (two), non-metal compounds (two), steroids (two), and two compounds were unclassified. There were forty-six compounds identified in ethanolic extracts of *T. obtusifolia* (Table 16), chemical classes with the most chemical compounds: sesquiterpenoids (seven), diterpenoids (five), fatty acids (five), methyl esters (three), steroids (three), fatty alcohols (two), resorcinols (two), and five compounds were unclassified.

## 3. Discussion

Most of the botanical extracts tested for their insecticidal activities proved to be effective repellents, feeding deterrents, and contact toxic against the *S. frugiperda* and *P. xylostella* larvae. Repellence, feeding deterrence, and contact toxicity of *E. capensis*, *T. dregeana, Turraea floribunda*, and *T. obtusifolia* extracts are recorded for the first time in this study. *Melia azedarach* was used as a positive control in this study as it is a well-known insecticidal plant in the Meliaceae family. The species has been proven to be an excellent insecticide against *S. frugiperda* [24,25,26,27,28,29,30,31]. In addition, *M. azedarach* extracts were found to be an effective botanical insecticide against *P. xylostella* in studies by Charleston et al. [32], Charleston et al. [12], Chen et al. [33], Chen et al. [34], Defagó et al. [35], Dilawari et al. [36], Dilawaxi et al. [37], Kumar et al. [38], Patil and Goud [39], Qiu et al. [40], Rani et al. [41], Sharma et al. [42], and Singh et al. [43].

Plant extracts with repellent activities are those with compounds that have irritating effects, causing insects to move away from them [44]. All plant extracts evaluated had repellence against the *S. frugiperda* and *P. xylostella* larvae, except for the ethanolic extracts of *T. floribunda* that had attractancy against the two tested larvae. However, there were interspecific differences as the botanical extracts were more susceptible as repellents to the *P. xylostella* larvae than to the *S. frugiperda* larvae (Table 1 and Table 2). Accordingly, seven extracts displayed repellency against *P. xylostella* larvae as follows: one in class V, three in IV, and five in III. In comparison, extracts displayed repellency against *S. frugiperda* according to the following level of activity: one in class V, two in IV, and one in III. It is not surprising that *M. azedarach* extracts were found to have the highest repellent activity against both *S. frugiperda* and *P. xylostella* larvae in this study, as this plant is known to have excellent repellent and insecticidal properties against several insect pests in several studies. Interestingly, *T. dregeana* recorded the same repellence activity as *M. azedarach* against both *S. frugiperda* and *P. xylostella* larvae. Against *S. frugiperda*, acetone extracts repelled 71% of the larvae (Table 1); meanwhile, against *P. xylostella*, acetone and ethanol extracts repelled 65% of the larvae (Table 2). *Trichilia dregeana* extracts in the study by Adinew [45] were found to have a highly positive protectant ability against *Sitophilus zeamais* Motschulsky (Maize weevil), which is also a major pest of maize similar to *S. frugiperda*. Extracts of *T. floribunda* moderately repelled the *S. frugiperda* larvae, while *E. capensis* and *T. obtusifolia* extracts recorded the lowest repellence activity.

Plants with antifeeding activities have compounds that, once consumed, cause the insects to stop feeding and eventually die due to starvation [44]. A study by Farag et al. [46] suggested that the plant extracts with feeding deterrence activity act as a stomach poison when ingested by insects. These feeding deterrent compounds could help reduce crop damage [18]. *Melia azedarach* aqueous extracts, followed by *T. obtusifolia* (aqueous and ethanol) and *T. floribunda* aqueous extracts, recorded the highest feeding deterrence activity against the *S. frugiperda* larvae. It does not come as a surprise that *Turraea* species recorded high feeding deterrence activity as in the study by Chimbe and Galley [47], another species of the genus, *Turraea nilotica* Kotschy & Peyr., was found to be effective against *Sitophilus oryzae* (rice weevil) larvae. Several studies also evaluated other species of the genus *Trichilia* for antifeedant properties. *Trichilia elegans* A.Juss. [48], *T. pallens* C.DC. [49], *T. pallida* Sw. [49], and *T. roka* (Forssk.) Chiov. [50] were found to have antifeedant activities against *S. frugiperda* larvae. Surprisingly, all *T. dregeana* extracts used in this study inhibited more *P. xylostella* larval feeding than *M. azedarach* extracts. This is contrary to the studies by Charleston et al. [32] and Dilawari et al. [36], where *M. azedarach* extracts recorded the highest antifeedant properties against the *P. xylostella* larvae. In this study, aqueous extracts of *T. obtusifolia* and ethanolic extracts of *E. capensis* also recorded high feeding deterrence activity against the *P. xylostella* larvae, with feeding deterrence coefficients of 86.74 and 85.79, respectively. The repellence activity of *E. capensis* coincides with that in the study by Champagne [51], in which the extracts acted as growth inhibitors and were toxic to *Peridroma saucia* Hübner (variegated cutworm) larvae. *Trichilia silvatica* C.DC. extracts were reported as good antifeedants against *P. xylostella* larvae [52].

Aqueous extracts of *E. capensis* and *T. floribunda* caused the highest *S. frugiperda* larval mortality, with recorded LC_50_ values of 0.14 mg/kg and 0.56 mg/kg, respectively. In the current study, all extracts of *M. azedarach* were less toxic to the *S. frugiperda* larvae (with an LC_50_ of 707.95 mg/kg). These results are contrary to the results obtained in the study by Bullangpoti et al. [26], where *M. azedarach* ethanolic extracts caused high mortality against the *S. frugiperda* with a recorded a lower LC_50_ value of 1.4 g L^−1^. *Ekebergia capensis* and *T. dregeana* extracts caused the least mortality to the larvae. However, in the study by Rioba and Stevenson [53], two members of the genus *Trichilia, T. pallens* C.DC., and *T. pallida* Sw., were found to cause high larval mortality against the *S. frugiperda* larvae. *Trichilia trijuga* Vell. extracts were found to be toxic to the *Crocidolomia binotalis* (cabbage cluster caterpillar) larvae [54] and *T. americana* (Sessé & Moc.) T.D.Penn. was toxic to the *Trichoplusia ni* (cabbage looper) and *Pseudaletia unipuncta* (armyworm moth) larvae [10]. On the other hand, all three extracts of *M. azedarach* and aqueous extracts of *T. obtusifolia* were more lethal to the *P. xylostella* larvae, with LC_50_ values of 0.14 mg/kg and 1.78 mg/kg, respectively. *Ekebergia capensis*, *T. dregeana*, and *T. floribunda* extracts caused insignificant toxicity against the *P. xylostella* larvae. This is contrary to this present study, as higher levels (LC_50_ value of 691.83 and 707.95 mg/kg) of the extracts of *T. dregeana* were needed to kill 50% of the larvae. *Trichilia emetica* methanol extracts resulted in high *P. xylostella* larval mortality with a recorded LC_50_ value of 0.94 mg.m^−1^ in the study by Munyemana and Alberto [55]. There may be a relationship between the toxicity against the *P. xylostella* of *Turraea* species screened in this study with the study of Essoung et al. [56] and Essoung et al. [57], where *T. floribunda* and other *Turraea* species *T. abyssinica* Hochst., *T. nilotica* Kotschy & Peyr., and *T. wakefieldii* Oliv. extracts were toxic to *Tuta obsoluta* (tomato leafminer) larvae. Growth inhibitory and toxicity activity of eight *Trichilia* species *T. americana* (Sesse & Mocino) Pennington, *T. connaroides* (Wright & Am.), *T. glabra* L., *T. havanensis* Jacq., T. *hirta* L., *T. martiana* C.DC, *T. pleeana* (A. Juss.) C.DC, and *T. quadrijuga* subsp. *cinerascens* (C.DC) Pennington extracts were evaluated on *Peridroma saucia* (variegated cutworm) and *Spodoptera litura* (cotton leafworm).

In the present work, ethanol extracts yielded the highest number of chemical compounds except for *T. floribunda*, where acetone extracts yielded 60 compounds, whereas ethanol yielded 30 compounds. This coincides with the antifeedant results, as ethanol extracts had better repellence, feeding deterrence, and contact toxicity than acetone extracts. Four chemical compounds were present in acetone and ethanol extracts of all five Meliaceae species studied: methyl alcohol, neophytadiene, phytol, and β-sitosterol. The tridecanoic acid methyl ester was present in all plant extracts except in ethanolic extracts of *T. dregeana*. The terpene derivatives phytol (present in all plant extracts in the current study) have been reported to have insecticidal [58] and pesticidal activities [59]. After all the chemical compounds identified in GC-MS analysis of plant extracts were classified, it was found that eight classes were common in all ethanol extracts. These were alcohols, diterpenoids, esters, fatty acids, fatty aldehydes, methyl esters, steroids, and triterpenoids. The five species’ most common classes in acetone extracts were alcohol, alkane, diterpenoid, ester, non-metal compound, organosilicon, and triterpenoid. All five plant species evaluated (either aqueous, acetone, or ethanol extracts) had repellence, feeding deterrence, and contact toxicity activity against *S. frugiperda* and *P. xylostella* larvae to some extent. The GC-MS analysis results strongly support these results as the two most well-known groups, phenols, and terpenes, known to have insecticidal and antifeedant properties, were present in all the plant extracts. *Trichilia dregeana* extracts exhibited excellent repellence activity and feeding deterrence against the two test larvae as the positive control, *M. azedarach* extracts. GC-MS analysis revealed that ethanol extracts of *T. dregeana* contained a high number of chemical classes that are terpenes (i.e., diterpenoid, sesquiterpenoid, triterpenoid, and steroid) and phenols (i.e., gingerdione). In acetone extracts of *T. dregeana*, four terpenes were identified (i.e., diterpenoid, sesquiterpenoid, triterpenoid, and steroid), as well as four phenols (i.e., 7-hydroxycoumarin, alkylbenzene, gingerdione, and resorcinol). Chemical compound phenol, 2,2′-methylenebis[6-(1,1-dimethylethyl)-4-methyl- identified in ethanolic extracts of *T. dregeana* was reported to have repellent, larvicidal, adulticidal, and oviposition deterrence activities against insects in a study by Chen et al. [60]. In studies by Curcino-Vieira et al. [61] and Tan and Luo [62], chemical compounds such as coumarins, diterpenes, flavonoids, glycosylated lignans, limonoids, monoterpenes, sesquiterpenes, steroids, and triterpenes isolated from the genus *Trichilia* were found to have insect feeding activities [63,64,65], and they may be toxic to insects [66,67]. *Ekebergia capensis* extracts exhibited good repellence, feeding deterrence, and contact toxicity against the test insects. GC-MS analysis revealed that ethanol extracts of *E. capensis* contained 50 compounds, of which 16 are terpenes belonging to diterpenoid, ergosterol, sesquiterpenoid, and steroid chemical classes. Conversely, acetone extracts identified thirty-three compounds, of which nine are terpenes (belonging to classes diterpenoid, sesquiterpenoid, and triterpenoid), and one is a phenol (cholesterol). The two members of the genus *Turraea, T. floribunda*, and *T. obtusifolia*, recorded minor activities in the antifeedant testing against the test insects. The presence of different chemical classes of compounds such as diterpenoids, flavonoids, limonoids, and terpenoids in some *Turraea* spp. have been associated with insecticidal activities in previous studies by Essoung et al. [56]; Ndung’u et al. [68]; Udenigwe et al. [69]; Yuan et al. [70]; Xu et al. [71]; and Zanin et al. [72]. Chemical groups other than phenols and terpenes, which have been recorded to have insecticidal, antifeedant, and insect repellent activities, have also been identified in the current study. For example, 9,12-otadecadienoic acid (Z,Z)- present in all *T. obtusifolia* extracts was reported to have insect-repellent properties in the study by Paulpriya et al. [59].

## 4. Materials and Methods

### 4.1. Antifeedant and Insecticidal Analysis

#### 4.1.1. Sample Preparations

Leaves were dried under shade at room temperature (25 °C), then ground into a fine powder using an electric grinder. Extraction was carried out according to the procedures of Warthen et al. [73], with some slight modifications. Ten grams of each powdered sample were extracted in 100 mL of water, acetone, and ethanol separately for 72 h at room temperature. After extraction, the solutions were filtered through Whatman No.40 filter paper, and the solvents were removed using a rotary evaporator. Methanol was used to dissolve the organic residues, where 0.5% and 1.0% solutions were prepared for each sample.

#### 4.1.2. Insects Selection and Rearing

*S. frugiperda* and *P. xylostella* second instar larvae strains (between 3 to 7 days old) were obtained from the Agricultural Research Council- Vegetable and Ornamental Plants (ARC- VOPI) in Pretoria, where they were reared, and the information on their age was also obtained.

#### 4.1.3. Repellence Bioassay

**Figure 1:**
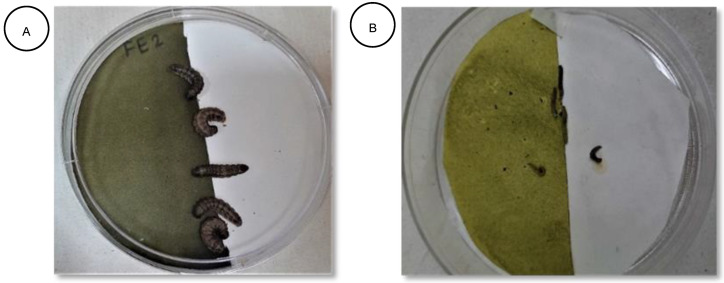
(**A**) Treated half and untreated half of filter paper with *S. frugiperda* larvae. (**B**) Treated half and untreated half of filter paper with *P. xylostella* larvae.

The repellence bioassay of the plant samples was assessed using Standard Method Number 3, described by McDonald et al. [74], with some modifications. Repellence tests were conducted using Whatman No.40 filter papers as opposed to the strips of aluminum foil laminated to 40 lb. kaft paper, as described in the study by McDonald et al. [74]. The substrata were prepared by cutting a filter paper in half and placing it in 0.5% and 1.0% solutions of the plant extracts for 1 min, and then allowing it to air dry at room temperature overnight. Each half of the treated disk was attached lengthwise, edge to edge, to an untreated half-disk of the filter paper with cellulose tape and placed in a petri dish (Figure 1A,B). To avoid cannibalism in a petri dish, five larvae of each insect were placed in the middle of each filter paper circle and covered. For five hours, at hourly intervals, individuals that settled on each half of the filter paper disk were counted, and the experimental design was run once. The average of the counts was converted to express the percentage repulsion (PR) as follows:

PR = 2 × (C − 50) (1)

where *C* is the percentage of insects on the untreated half of the disk. Positive percentage repulsion values expressed repellence, and negative percentage repulsion values expressed attractancy. The averages of the percentage repulsion were then assigned different classes using the scale as follows [75] (Table 17):

#### 4.1.4. Feeding Deterrence Test

**Figure 2:**
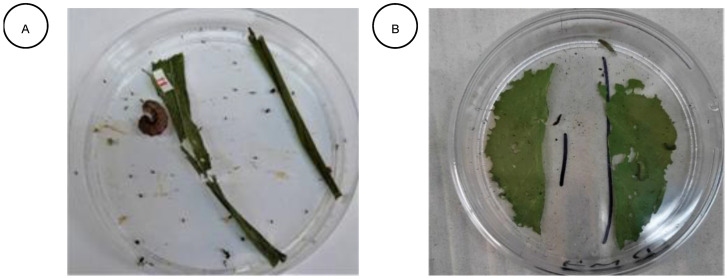
(**A**) Maize leaf disk for the feeding deterrence assay against *S. frugiperda* after 24 h. (**B**) Cabbage leaf disk for the feeding deterrence assay against *P. xylostella* after 24 h.

The potency of the feeding deterrence effect of plant leaf extracts against *S. frugiperda* and *P. xylostella* was determined by using the leaf disk bioassay. Maize and cabbage leaves were used as the test food for *S. frugiperda* and *P. xylostella*, respectively. The leaves were soaked in either water only (control leaf disks K) or in a 1% plant extract solution of aqueous, acetone, and ethanol separately (treated leaf disks E). The leaf disks (Figure 2A,B) were allowed to air dry at room temperature for about 30 min and weighed before they were presented to the larvae in petri dishes for 24 h, during which they were serving as the sole food source. The feeding behaviour of the larvae was recorded under three different conditions: (1) pure food, which comprised two control leaves (KK) (control test); (2) food with one control leaf (K) and one treated leaf (E) (choice test); and (3) food with two treated leaves (EE) (no choice test). After 24 h, the remaining leaves were reweighed, and mean percentages of feeding deterrence (FD) were calculated for each plant extract based on the weight of leaves before and after the tests. FD was calculated as follows:

FD = (C − T/C + T) × 100 (2)

C = weight of control leaves; T = weight of treated leaves.

After the FD values were calculated, three coefficients for the feeding deterrent activity from all three tests for each plant extract were calculated as follows [75]:

1. Absolute deterrence coefficient

A = (KK − EE/KK + EE) × 100(3)

2. Relative deterrence coefficient

R = (K − E/K + E) × 100(4)

3. Total deterrence coefficient

T = A + R(5)

Values of the total deterrence coefficient (A) served as an index of the feeding deterrence activity which was expressed on a scale between 0 and 200. Plant extracts with a total deterrence coefficient of between 150–200 were marked ++++; 100–150, +++; 50–100, ++, and 0–50 + [75].

#### 4.1.5. Topical Application Bioassay

The topical treatment assay tested the direct contact toxicity of the plant extracts, using Standard Method Number 1 described by McDonald et al. [74] with some modifications. Plant extract solutions of 0.5% and 1% were used for this test. Larvae were chilled for 10 min instead of being anesthetized with carbon dioxide in a Buchner funnel for about 5 min, as described in the study by McDonald et al. [74]. The immobilized larvae were picked up individually with forceps. Ten microliters of each plant extract solution were applied to the dorsum of each larva. Five larvae were treated at each dose and then transferred to a petri dish. After 24 h, the larvae were examined, and those that did not respond to gentle touch were considered dead. The number of dead larvae was recorded, and corrected mortality rates were calculated using the formula:

Percent larval mortality = (number of dead larvae/total number of treated larvae) × 100 (6)

Probit analysis [76] was used to analyse concentration-mortality data.

### 4.2. Gas Chromatography-Mass Spectrometry (GC-MS) Analysis

GC-MS analysis was used to identify chemical compounds present in all five selected Meliaceae species. The patterns of the mass spectra fragmentation and their retention indices were compared with the ones stored in the computer library to identify the chemical components found in the plant extracts [77].

#### 4.2.1. Sample Preparation

One gram of powdered samples was extracted in acetone and ethanol for 24 h at room temperature. The extracts were centrifuged at 13,000× *g* rpm for 10 min at 10 °C. Whatman No.1 filter paper was used to filter the solutions, and a rotary evaporator was used to evaporate or concentrate the solvents. One milliliter of methanol was used to dissolve the organic residues. The solutions were transferred into dark amber vials using syringe filters.

#### 4.2.2. Gas Chromatography-High-Resolution-Time-of-Flight Mass Spectrometry (GC-HRTOF- MS) Analyses

The samples were analysed on the GC-HRTOF- MS system equipped with an Agilent 7890A gas chromatograph (Agilent Technologies, Inc., Wilmington, DE, USA). This system operates in high-resolution, equipped with a Gerstel MPS multipurpose autosampler (Gerstel Inc., Germany) and capillary column (Rxi- 5 ms- 30 m × 0.25 mm ID × 0.25 µm). For each plant extract, a volume of 1 µL was injected in a spitless mode. The program was started at 70 °C, held for 0.5 min, ramped at 10 °C/min to 150 °C, held for 2 min, ramped at 10 °C/min to 330 °C, and held for 3 min for the column to bake out. The samples were analysed at an MS data acquisition rate of 13 spectra/s, m/z range of 30–1000, electron ionization at 70 eV, ion source temperature was set at 250 °C, and the system extraction frequency was set at 1.25 kHz. Solvent blanks were also used to observe for contamination and impurities. Compounds were identified by matching the generated spectra with the NIST, Mainlib, and Feihn reference library databases on ChromaTOF-HRT^®^ (LECO Corporation, St. Joseph, MI, USA). Subsequent retention time alignment, matched filtration, peak picking, detection, and matching were conducted on a data station equipped with the ChromaTOF-HRT^®^ software (LECO Corporation, St. Joseph, MI, USA). Parameters adopted for processing included a signal-to-noise ratio (S/N) of 100, a similarity match above 70%, and data presented in Table 7, Table 8, Table 9, Table 10, Table 11, Table 12, Table 13, Table 14, Table 15 and Table 16 representing only compounds occurring at least twice in triplicate injections. The collected GC-HRTOF-MS dataset was converted to mzML format using the LECO ChromaTOF-HRT software and then processed (peak picking and alignment) on the XCMS open-source tool.

## 5. Conclusions

Meliaceae species are abundant large tree species, so they would be suitable to supply very large-scale production of botanical insecticides; thus, their potential use in controlling insect pests is promising. This study provides potential evidence that further confirms the findings of many previous reports that Meliaceae members can be used as repellents, insecticides, and antifeedants to control *S. frugiperda* and *P. xylostella* insects, two of the most important agricultural pests that mostly attack crops which form part of the primary staple food. All extracts of the five evaluated species indicated repellence to the *S. frugiperda* and *P. xylostella* larvae, except for the ethanolic extracts of *T. floribunda*, which showed attraction to both the larvae. All extracts evaluated exhibited feeding deterrence to the *S. frugiperda* and *P. xylostella* larvae to some extent, except for the ethanol extracts of *T. floribunda*, which had inert antifeeding compounds. Aqueous extracts of *E. capensis* and *T. floribunda* were more toxic to the *S. frugiperda* larvae, and all extracts of *M. azedarach* were more toxic to the *P. xylostella* larvae. The GC-MS analysis results strongly support the insecticidal activities of the evaluated extracts as the two most well-known groups, phenols and terpenes, known to have insecticidal and antifeedant properties, were present in all the plant extracts. Therefore, this further corroborates the recorded traditional uses of these plants as insecticides and antifeedants. Plants that have indicated the most promising results are *E. capensis*, *T. floribunda*, and *T. obtusifolia*. These plants should be subjected to further quantitative phytochemical studies focusing on isolating and identifying active compounds rather than simply screening the plant extracts for insecticidal and antifeedant activity, as plant extracts may contain many compounds along with those that may cause negative side effects and toxicity. Further research should also be conducted regarding their safe use and non-target effects, and to determine if they can maintain yield at comparable levels to synthetic pesticides. Field trial evaluations of insecticidal and antifeedant plant extracts may also need to be undertaken to assess their impact on crop yield and damage and evaluate insect resistance issues in comparison to synthetic pesticides. This study’s results are significant as they will generate new and alternative natural products that can help improve biological effectiveness, lower residuals, increase nontoxic agricultural products, and decrease their presence in foods.

## Figures and Tables

**Table 1:** Average repellence of five Meliaceae species leaf extracts against *Spodoptera frugiperda* larvae using the treated filter paper test.

**Plant Species**	**Extract**	**Dose/** **Concentration (%)**	**Percentage Repulsion (PR) = 2 × (C − 50) in Hours** C = Is the Percentage of Insects on the Untreated Half of the Disk				**Average (PR)**	**Class**
			**1 H**	**2 H**	**3 H**	**4 H**		
1. *Ekebergia capensis* Sparrm	Aqueous	0.5	60	60	0	100	33	II
		1.0	−20	20	20	20		
	Acetone	0.5	−20	20	20	20	15	I
		1.0	20	20	60	−20		
	Ethanol	0.5	60	20	20	20	10	I
		1.0	−60	−20	20	20		
2. *Melia azedarach* L.	Aqueous	0.5	100	100	100	100	95	V
		1.0	100	60	100	100		
	Acetone	0.5	20	60	0	0	28	II
		1.0	60	−20	100	0		
	Ethanol	0.5	20	100	20	20	65	IV
		1.0	100	100	60	100		
3. *Trichilia dregeana* Harv. & Sond.	Aqueous	0.5	20	20	20	20	40	II
		1.0	20	60	60	100		
	Acetone	0.5	60	60	50	0	71	IV
		1.0	100	100	100	100		
	Ethanol	0.5	100	60	−60	−60	30	II
		1.0	20	60	60	60		
4. *Turraea floribunda* Hochst.	Aqueous	0.5	−60	100	100	100	49	III
		1.0	0	100	0	50		
	Acetone	0.5	60	60	20	100	20	I
		1.0	100	−100	−60	−20		
	Ethanol	0.5	−60	−100	−60	−100	−55	-
		1.0	−60	−60	20	−20		
5. *Turraea obtusifolia* Hochst.	Aqueous	0.5	0	0	5	0	3	I
		1.0	−20	−20	20	20		
	Acetone	0.5	60	−20	60	−20	5	I
		1.0	−20	−20	−20	20		
	Ethanol	0.5	−20	20	20	20	30	II
		1.0	20	60	60	60		

**Table 2:** Average repellence of five Meliaceae species leaf extracts against *Plutella xylostella* larvae using the treated filter paper test.

**Plant Species**	**Extract**	**Dose/** **Concentration (%)**	**Percentage Repulsion (PR) = 2 × (C − 50) in Hours** C = Is the Percentage of Insects on the Untreated Half of the Disk				**Average (PR)**	**Class**
			**1 h**	**2 h**	**3 h**	**4 h**		
1. *Ekebergia capensis*	Aqueous	0.5	60	60	100	100	60	III
		1.0	−20	20	60	100		
	Acetone	0.5	−20	20	60	100	50	III
		1.0	20	20	100	100		
	Ethanol	0.5	60	20	100	100	50	III
		1.0	−60	−20	100	100		
2. *Melia azedarach*	Aqueous	0.5	100	100	100	100	90	V
		1.0	100	60	100	60		
	Acetone	0.5	20	60	20	20	35	II
		1.0	60	−20	60	60		
	Ethanol	0.5	20	100	100	60	80	IV
		1.0	100	100	100	60		
3. *Trichilia dregeana*	Aqueous	0.5	20	20	60	60	45	III
		1.0	20	60	60	60		
	Acetone	0.5	60	60	60	20	65	IV
		1.0	100	100	60	60		
	Ethanol	0.5	100	60	100	60	65	IV
		1.0	20	60	60	60		
4. *Turraea floribunda*	Aqueous	0.5	−60	100	20	60	43	III
		1.0	0	100	100	20		
	Acetone	0.5	60	60	20	60	30	II
		1.0	100	−100	20	20		
	Ethanol	0.5	−60	−100	60	60	−10	-
		1.0	−60	−60	20	60		
5. *Turraea obtusifolia*	Aqueous	0.5	0	0	60	60	15	I
		1.0	−20	−20	20	20		
	Acetone	0.5	60	−20	20	60	30	II
		1.0	−20	−20	60	100		
	Ethanol	0.5	20	−60	60	100	31	II
		1.0	60	−50	20	100		

**Table 3:** Feeding deterrent activity coefficient of five Meliaceae species leaf extracts against *Spodoptera frugiperda*.

Plant Species	Extract	Coefficient of Deterrence			Efficacy of Extracts
		Absolute (A)	Relative (R)	Total (T)	
1. *Ekebergia capensis*	Aqueous	21.66	26.61	48.27	+
	Acetone	25.14	19.97	45.11	+
	Ethanol	−10.77	28.55	17.78	+
2. *Melia azedarach*	Aqueous	48.04	35.88	83.92	++
	Acetone	58.14	3.43	61.57	++
	Ethanol	28.96	8.39	37.35	+
3. *Trichilia dregeana*	Aqueous	29.95	32.07	62.02	++
	Acetone	29.01	23.84	52.85	++
	Ethanol	14.30	31.99	46.29	+
4. *Turraea floribunda*	Aqueous	24.40	42.56	66.96	++
	Acetone	17.86	3.04	20.90	+
	Ethanol	3.04	−15.93	−12.89	0
5. *Turraea obtusifolia*	Aqueous	40.91	26.38	67.29	++
	Acetone	17.51	17.17	34.65	+
	Ethanol	27.06	41.38	68.44	++

**Table 4:** Feeding deterrent activity coefficient of five Meliaceae species leaves extracts against *Plutella xylostella* larvae.

Plant Species	Extract	Coefficient of Deterrence			Efficacy of Extract
		Absolute (A)	Relative (R)	Total (T)	
1. *Ekebergia capensis*	Aqueous	27.65	3.61	31.26	+
	Acetone	50.60	2.57	53.17	++
	Ethanol	40.39	45.40	85.79	++
2. *Melia azedarach*	Aqueous	34.79	3.34	38.13	+
	Acetone	32.89	13.55	46.44	+
	Ethanol	56.13	4.13	60.26	++
3. *Trichilia dregeana*	Aqueous	45.85	52.92	98.77	++
	Acetone	62.37	49.88	112.25	+++
	Ethanol	63.52	35.87	99.39	++
4. *Turraea floribunda*	Aqueous	34.70	−20.75	13.95	+
	Acetone	49.41	−23.48	25.93	+
	Ethanol	49.65	−5.43	44.22	+
5. *Turraea obtusifolia*	Aqueous	42.24	44.50	86.74	++
	Acetone	38.94	0.94	39.88	+
	Ethanol	55.43	−1.06	54.37	++

**Table 5:** Toxicity of five Meliaceae species leaf extracts applied topically to *Spodoptera frugiperda* larvae.

Plant Species	Extracts	Concentration (ppm)	log10 (Concentration)	% Dead	Probit	LD_50_ (mg/kg)
1. *Ekebergia capensis*	Aqueous	500	2.70	80	5.84	0.14
		1000	3.00	80	5.84	
	Acetone	500	2.70	80	5.84	707.95
		1000	3.00	20	4.16	
	Ethanol	500	2.70	20	4.16	851.14
		1000	3.00	60	5.25	
2. *Melia azedarach*	Aqueous	500	2.70	20	4.16	707.95
		1000	3.00	80	5.84	
	Acetone	500	2.70	80	5.84	707.95
		1000	3.00	20	4.16	
	Ethanol	500	2.70	80	5.84	707.95
		1000	3.00	20	4.16	
3. *Trichilia dregeana*	Aqueous	500	2.70	20	4.16	707.95
		1000	3.00	80	5.84	
	Acetone	500	2.70	60	5.25	707.95
		1000	3.00	40	4.75	
	Ethanol	500	2.70	40	4.75	588.84
		1000	3.00	80	5.84	
4. *Turraea floribunda*	Aqueous	500	2.70	60	5.25	0.56
		1000	3.00	60	5.25	
	Acetone	500	2.70	80	5.84	707.95
		1000	3.00	20	4.16	
	Ethanol	500	2.70	20	4.16	6.92
		1000	3.00	20	4.16	
5. *Turraea obtusifolia*	Aqueous	500	2.70	60	5.25	371.54
		1000	3.00	80	5.84	
	Acetone	500	2.70	40	4.75	707.95
		1000	3.00	60	5.25	
	Ethanol	500	2.70	60	5.25	371.54
		1000	3.00	80	5.84	

**Table 6:** Toxicity of five Meliaceae species leaf extracts applied topically to *Plutella xylostella* larvae.

Plant Species	Extracts	Concentration (ppm)	log10 (Concentration)	% Dead	Probit	LD_50_ (mg/kg)
1. *Ekebergia capensis*	Aqueous	500	2.70	40	4.75	691.83
		1000	3.00	60	5.25	
	Acetone	500	2.70	20	4.16	707.95
		1000	3.00	80	5.84	
	Ethanol	500	2.70	40	4.75	691.83
		1000	3.00	60	5.25	
2. *Melia azedarach*	Aqueous	500	2.70	80	5.84	0.14
		1000	3.00	80	5.84	
	Acetone	500	2.70	80	5.84	0.14
		1000	3.00	80	5.84	
	Ethanol	500	2.70	80	5.84	0.14
		1000	3.00	80	5.84	
3. *Trichilia dregeana*	Aqueous	500	2.70	40	4.75	691.83
		1000	3.00	60	5.25	
	Acetone	500	2.70	80	5.84	707.95
		1000	3.00	20	4.16	
	Ethanol	500	2.70	60	5.25	691.83
		1000	3.00	40	4.75	
4. *Turraea floribunda*	Aqueous	500	2.70	80	5.84	851.14
		1000	3.00	40	4.75	
	Acetone	500	2.70	20	4.16	851.14
		1000	3.00	60	5.25	
	Ethanol	500	2.70	20	4.16	707.95
		1000	3.00	80	5.84	
5. *Turraea obtusifolia*	Aqueous	500	2.70	40	4.75	1.78
		1000	3.00	40	4.75	
	Acetone	500	2.70	20	4.16	1318.26
		1000	3.00	40	4.75	
	Ethanol	500	2.70	80	5.84	1318.26
		1000	3.00	60	5.25	

**Table 7:** Compounds identified in leaf acetone extracts of *Ekebergia capensis*.

RT (min)	Observed Ion *m*/*z*	MF	Name	MC	FC
1. 13.95	218.9578	C_15_H_24_O	(1R,3E,7E,11R)-1,5,5,8-Tetramethyl-12-oxabicyclo[9.1.0]dodeca-3,7-diene	Epoxide	26,6925.50
2. 29.96	263.8374	C_13_H_20_N_2_SSi	1,2-Benzisothiazol-3-amine, TBDMS derivative	Sugar	18,674.71
3. 13.45	202.1718	C_15_H_22_	1,8-Cyclopentadecadiyne	Sesquiterpenoid	115,106.00
4. 12.71	180.1142	C_11_H_16_O_2_	2(4H)-Benzofuranone, 5,6,7,7a-tetrahydro-4,4,7a-trimethyl-	Benzofuran	132,297.50
5. 16.78	165.1639	C_13_H_26_O	2-Undecanone, 6,10-dimethyl-	Fatty aldehyde	204,579.33
6. 30.25	326.7937	C_24_H_36_O_2_Si_2_	4-Methyl-2,4-bis(p-hydroxyphenyl)pent-1-ene, 2TMS derivative	Bisphenol A	25,467.00
7. 18.36	218.9095	C_20_H_40_	5-Eicosene, (E)-	Aliphatic hydrocarbon	70,769.00
8. 21.85	218.9260	C_18_H_35_NO	9-Octadecenamide, (Z)-	Fatty amide	448,270.50
9. 20.26	130.9614	C_11_H_16_FNO_3_	Benzeneethanamine, 2-fluoro-β,3,4-trihydroxy-N-isopropyl-	Organofluorine compound	173,648.67
10. 24.10	130.9736	C_39_H_28_O_4_	Bis[2-(cinnamoyloxy)-1-naphthyl]methane		10,127.50
11. 13.56	218.9391	C_15_H2_4_O	Caryophyllene oxide	Sesquiterpenoid	411,365.67
12. 27.33	394.3601	C_27_H_44_O	Cholesta-4,6-dien-3-ol, (3β)-	Cholesterol	108,829.33
13. 28.04	218.9138	C_6_H_18_O_3_Si_3_	Cyclotrisiloxane, hexamethyl-	Organosilicon	24,262.25
14. 22.87	155.1795	C_27_H_56_	Heptacosane	Alkane	215,283.20
15. 13.54	218.8042	C_16_H_34_	Hexadecane	Alkane	729,031.09
16. 2.59	32.0408	H_4_N_2_	Hydrazine	Non-metal compound	12,709.83
17. 29.62	426.3880	C_30_H_50_O	Lupeol	Triterpenoid	84,095.50
18. 2.96	32.0260	CH_4_O	Methyl Alcohol	Alcohol	2773,625.86
19. 18.18	256.2399	C_16_H_32_O_2_	n-Hexadecanoic acid	Fatty acid	829,242.33
20. 16.70	218.9267	C_20_H_38_	Neophytadiene	Diterpenoid	202,795.80
21. 21.89	154.1226	C_9_H_19_NO	Nonanamide	Amide	349,356.50
22. 24.39	218.7771	C_28_H_5_8	Octacosane	Alkane	171,462.67
23. 29.30	408.3768	C_32_H_52_O_2_	Olean-12-en-3-ol, acetate, (3β)-	Triterpenoid	210,758.00
24. 17.09	224.0999	C_22_H_23_NO_4_	Phthalic acid, 4-cyanophenyl heptyl ester	Ester	454,868.00
25. 23.33	218.8161	C_20_H_30_O_4_	Phthalic acid, heptyl 3-methylbutyl ester	Ester	4577,088.00
26. 16.70	218.8513	C_20_H_40_O	Phytol	Diterpenoid	202,160.25
27. 14.32	218.8335	C_15_H_24_O	Tetracyclo[6.3.2.0(2,5).0(1,8)]tridecan-9-ol, 4,4-dimethyl-	No records	91,373.50
28. 17.65	227.2006	C_14_H_28_O_2_	Tridecanoic acid, methyl ester	Methyl ester	380,039.50
29. 30.36	283.8030	C_18_H_45_AsO_3_Si_3_	Tris(tert-butyldimethylsilyloxy)arsane	Ester	29,655.00
30. 19.68	199.1691	C_12_H_24_O_2_	Undecanoic acid, methyl ester	Methyl ester	74,944.00
31. 14.46	200.1558	C_15_H_20_	α-Calacorene	Sesquiterpenoid	49,402.00
32. 27.58	431.3842	C_31_H_52_O_3_	α-Tocopheryl acetate	Triterpenoid	183,414.80
33. 28.36	401.3731	C_31_H_52_O_2_	β-Sitosterol acetate	Triterpenoid	696,117.67

**Table 8:** Compounds identified in leaf ethanol extracts of *Ekebergia capensis*.

RT (min)	Observed Ion *m*/*z*	MF.	Name	MC	FC
1. 13.49	218.9559	C_15_H_24_O	(-)-Spathulenol	Sesquiterpenoid	225,729.33
2. 23.93	150.1032	C_12_H_15_ClN_2_	(1R,2R,4S)-2-(6-Chloropyridin-3-yl)-7-methyl-7-azabicyclo[2.2.1]heptane	Epibatidine analogues	148,243.33
3. 13.98	220.1821	C_15_H_24_O	(1R,3E,7E,11R)-1,5,5,8-Tetramethyl-12-oxabicyclo[9.1.0]dodeca-3,7-diene	Epoxide	499,685.75
4. 29.29	263.9630	C_13_H_20_N_2_SSi	1,2-Benzisothiazol-3-amine, TBDMS derivative	Sugar	23,401.00
5. 14.34	218.7645	C_15_H_24_O	10,10-Dimethyl-2,6-dimethylenebicyclo[7.2.0]undecan-5β-ol		193,820.25
6. 19.99	265.2496	C_21_H_36_O_2_	11,14,17-Eicosatrienoic acid, methyl ester	Methyl ester	815,008.50
7. 12.74	180.1142	C_11_H_16_O_2_	2(4H)-Benzofuranone, 5,6,7,7a-tetrahydro-4,4,7a-trimethyl-	Benzofuran	260,204.00
8. 4.15	110.0360	C_6_H_6_O_2_	2-Furancarboxaldehyde, 5-methyl-	Aryl-aldehyde	76,995.50
9. 4.59	112.0154	C_5_H_4_O_3_	2H-Pyran-2,6(3H)-dione	Valerolactone	166,899.00
10. 8.89	150.0679	C_9_H_10_O_2_	2-Methoxy-4-vinylphenol	Ketone	215,229.67
11. 5.13	102.0550	C_6_H_13_NO	2-Pyrrolidinemethanol, 1-methyl-	Proline	1586,572.00
12. 16.80	193.1957	C_13_H_26_O	2-Undecanone, 6,10-dimethyl-	Fatty aldehyde	441,117.67
13. 17.00	278.2967	C_20_H_40_O	3,7,11,15-Tetramethyl-2-hexadecen-1-ol	Diterpenoid	321,637.00
14. 14.96	218.7685	C_20_H_27_FO_2_	3-Fluorobenzoic acid, tridec-2-ynyl ester	Organofluorine compound	255,232.00
15. 6.58	144.0415	C_6_H_8_O_4_	4H-Pyran-4-one, 2,3-dihydro-3,5-dihydroxy-6-methyl-	Fatty acid	1257,832.00
16. 13.21	200.1558	C_15_H_20_	4-Isopropyl-6-methyl-1-methylene-1,2,3,4-tetrahydronaphthalene	Sesquiterpenoid	67,046.83
17. 28.47	340.8050	C_24_H_36_O_2_Si_2_	4-Methyl-2,4-bis(p-hydroxyphenyl)pent-1-ene, 2TMS derivative	Bisphenol A	17,941.50
18. 15.56	218.8078	C_13_H_20_O_2_	6,6-Dimethyl-2-(3-oxobutyl)bicyclo[3.1.1]heptan-3-one	Oxepane	360,494.00
19. 16.14	196.1090	C_11_H_16_O_3_	6-Hydroxy-4,4,7a-trimethyl-5,6,7,7a-tetrahydrobenzofuran-2(4H)-one	Benzofuran	480,778.00
20. 15.70	180.0778	C_15_H_26_O_2_	7-Acetyl-2-hydroxy-2-methyl-5-isopropylbicyclo[4.3.0]nonane	Sesquiterpenoid	296,149.33
21. 21.89	282.2742	C_18_H_35_NO	9-Octadecenamide, (Z)-	Fatty amide	875,563.20
22. 10.36	122.0361	C_7_H_6_O_2_	Benzaldehyde, 4-hydroxy-	Hydroxybenzaldehyde	78,283.50
23. 28.39	400.3717	C_28_H_48_O	Campesterol	Ergosterol	683,575.33
24. 14.80	218.9483	C_15_H_24_O	Caryophylla-4(12),8(13)-dien-5α-ol	Sesquiterpenoid	309,195.00
25. 13.60	220.1821	C_15_H_24_O	Caryophyllene oxide	Sesquiterpenoid	768,061.33
26. 8.55	110.0361	C_6_H_6_O_2_	Catechol	Catechol	198,727.00
27. 27.36	379.3372	C_27_H_44_O	Cholesta-4,6-dien-3-ol, (3β)-	Cholesterol	293,677.67
28. 13.34	218.7878	C_12_H_7_Cl_5_O_4_	Fumaric acid, ethyl pentachlorophenyl ester	Ester	166,528.00
29. 3.38	97.0278	C_4_H_8_O_4_	Glycolaldehyde dimer	Pentose	8,931.50
30. 20.31	226.2170	C_16_H_33_NO	Hexadecanamide	Fatty amide	594,910.67
31. 23.05	258.2501	C_19_H_38_O_4_	Hexadecanoic acid, 2-hydroxy-1-(hydroxymethyl)ethyl ester	1-monoacylglycerol	184,585.67
32. 2.60	32.0564	H_4_N_2_	Hydrazine	Non-metal compound	24,186.67
33. 2.97	32.0261	CH_4_O	Methyl Alcohol	Alcohol	1931,798.17
34. 14.84	198.1405	C_15_H_18_	Naphthalene, 1,6-dimethyl-4-(1-methylethyl)-	Sesquiterpenoid	72,764.67
35. 16.72	278.2967	C_20_H_38_	Neophytadiene	Diterpenoid	454,427.18
36. 18.26	256.0055	C_16_H_32_O_2_	n-Hexadecanoic acid	Fatty acid	2543,102.33
37. 6.28	85.0840	C_6_H_13_NO	N-Methyl-L-prolinol	Amino acid	957,665.00
38. 20.13	284.2714	C_18_H_36_O_2_	Octadecanoic acid	Fatty acid	650,507.00
39. 12.24	206.1660	C_14_H_22_O	Phenol, 3,5-bis(1,1-dimethylethyl)-	Sesquiterpenoid	100,587.50
40. 18.16	279.1556	C_20_H_30_O_4_	Phthalic acid, heptyl pentyl ester	Ester	7104,005.33
41. 19.61	278.2963	C_20_H_40_O	Phytol	Diterpenoid	714,988.60
42. 28.60	412.3707	C_29_H_48_O	Stigmasterol	Steroid	825,673.33
43. 15.83	228.2081	C_14_H_28_O_2_	Tetradecanoic acid	Fatty acid	274,646.67
44. 18.28	213.1522	C_13_H_26_O_2_	Tridecanoic acid	Fatty acid	5679,271.50
45. 17.67	228.2043	C_14_H_28_O_2_	Tridecanoic acid, methyl ester	Methyl ester	307,666.00
46. 17.69	143.0654	C_12_H_24_O_2_	Undecanoic acid, methyl ester	Methyl ester	255,331.00
47. 12.89	200.1555	C_15_H_20_	α-Calacorene	Sesquiterpenoid	60,816.33
48. 27.60	431.3854	C_31_H_52_O_3_	α-Tocopheryl acetate	Triterpenoid	681,154.00
49. 29.34	426.3876	C_30_H_50_O	β-Amyrin	Triterpenoid	390,576.00
50. 29.01	414.3870	C_29_H_50_O	β-Sitosterol	Triterpenoid	1529,284.00

**Table 9:** Compounds identified in leaf acetone extracts of *Melia azedarach*.

RT (min)	Observed Ion *m*/*z*	MF	Name	MC	FC
1. 30.53	248.8762	C_11_H_10_O_6_	1,2,4-Benzenetricarboxylic acid, 1,2-dimethyl ester	Benzoic acid	21,666.00
2. 30.13	265.2098	C_13_H_20_N_2_SSi	1,2-Benzisothiazol-3-amine, TBDMS derivative	Sugar	23,300.33
3. 28.24	208.9309	C_12_H_22_Si_2_	1,2-Bis(trimethylsilyl)benzene	Organosilicon	11,527.50
4. 16.78	218.8432	C_14_H_28_O	2-Tetradecanone	Ketone	182,214.50
5. 21.78	218.8388	C_21_H_40_O_2_	4,8,12,16-Tetramethylheptadecan-4-olide	Beta-diketone	73,950.50
6. 30.24	258.8419	C_24_H_36_O_2_Si_2_	4-Methyl-2,4-bis(p-hydroxyphenyl)pent-1-ene, 2TMS derivative	Bisphenol A	56,309.00
7. 14.92	218.9359	C_14_H_20_O_3_	8-(2-Acetyloxiran-2-yl)-6,6-dimethylocta-3,4-dien-2-one	Fatty alcohol ester	151,894.00
8. 23.33	168.0373	C_24_H_38_O_4_	Bis(2-ethylhexyl) phthalate	Ester	54,139.00
9. 28.57	394.3614	C_29_H_46_	Cholesta-6,22,24-triene, 4,4-dimethyl-	Cholesterol	449,205.00
10. 28.25	207.9947	C_6_H_18_O_3_Si_3_	Cyclotrisiloxane, hexamethyl-	Organosilicon	22,319.83
11. 20.34	218.9400	C_27_H_56_	Heptacosane	Alkane	136,232.20
12. 18.43	218.9511	C_16_H_34_	Hexadecane	Alkane	461,468.60
13. 2.59	32.0036	H_4_N_2_	Hydrazine	Non-metal compound	31,792.00
14. 2.93	33.0194	H_3_NO	Hydroxylamine	Amine	687,694.00
15. 24.40	206.8313	C_20_H_42_O	Isobutyl hexadecyl ether	Ether	99,861.00
16. 15.46	218.8891	C_19_H_18_F_2_O_4_	Isophthalic acid, 3,5-difluorophenyl pentyl ester	Ester	9,181.00
17. 2.97	32.0260	CH_4_O	Methyl Alcohol	Alcohol	2893,853.89
18. 16.70	218.8535	C_20_H_38_	Neophytadiene	Diterpenoid	212,138.00
19. 20.24	130.9100	C_9_H_19_NO	Nonanamide	Amide	100,542.80
20. 19.59	278.2969	C_20_H_40_O	Phytol	Diterpenoid	354,182.00
21. 17.66	227.2007	C_14_H_28_O_2_	Tridecanoic acid, methyl ester	Methyl ester	263,243.33
22. 30.06	340.7472	C_18_H_45_AsO_3_Si_3_	Tris(tert-butyldimethylsilyloxy)arsane	Ester	21,078.67
23. 19.68	218.8605	C_12_H_24_O_2_	Undecanoic acid, methyl ester	Methyl ester	67,474.00
24. 29.63	432.3895	C_31_H_52_O_3_	α-Tocopheryl acetate	Triterpenoid	222,825.33
25. 28.37	401.3752	C_31_H_52_O_2_	β-Sitosterol acetate	Triterpenoid	548,590.25

**Table 10:** Compounds identified in leaf ethanol extracts of *Melia azedarach*.

RT (min)	Observed Ion *m*/*z*	MF	Name	MC	FC
1. 14.94	218.9352	C_13_H_16_O_4_	1,6,6-Trimethyl-7-(3-oxobut-1-enyl)-3,8-dioxatricyclo[5.1.0.0(2,4)]octan-5-one	Ketone	208,378.00
2. 17.00	138.1403	C_16_H_30_	1-Hexadecyne	Hydrocarbon	146,746.50
3. 17.00	278.2964	C_18_H_34_	1-Octadecyne	Hydrocarbon	182,460.00
4. 2.77	43.0049	C_2_H5ClO	2-Chloroethanol	Chloroethanol	10,825.00
5. 12.38	155.0941	C_8_H_13_NO_2_	2-Hydroxy-1-(1′-pyrrolidiyl)-1-buten-3-one	No record	163,420.00
6. 16.79	218.8744	C_14_H_28_O	2-Tetradecanone	Ketone	191,685.50
7. 16.79	218.8549	C_13_H_26_O	2-Undecanone, 6,10-dimethyl-	Fatty aldehyde	177,821.00
8. 16.94	40.9534	C_2_H_4_N_2_	3-Methyl-1,2-diazirine	No record	10,163.33
9. 16.30	144.0416	C_6_H_8_O_4_	4H-Pyran-4-one, 2,3-dihydro-3,5-dihydroxy-6-methyl-	Fatty acid	419,184.67
10. 21.87	218.9503	C_18_H_35_NO	9-Octadecenamide, (Z)-	Fatty amide	368,824.25
11. 12.30	220.1819	C_15_H_24_O	Butylated Hydroxytoluene	Phenylpropane	26,415.67
12. 28.39	405.0388	C_31_H_52_O_3_	Cholesterol 3-O-[[2-acetoxy]ethyl]-	No record	498,714.50
13. 29.66	430.3826	C_29_H_50_O_2_	dl-α-F	Resorcinol	505,469.00
14. 20.27	130.9692	C_12_H_25_NO	Dodecanamide	Fatty amide	74,969.00
15. 13.24	128.0426	C_12_H_7_Cl_5_O_4_	Fumaric acid, ethyl pentachlorophenyl ester	Ester	90,985.50
16. 20.27	130.9721	C_16_H_33_NO	Hexadecanamide	Fatty amide	107,671.50
17. 2.90	32.0228	CH_4_O	Methyl Alcohol	Alcohol	1607,627.00
18. 13.03	157.1220	C_10_H_20_O_2_	n-Decanoic acid	Fatty acid	86,722.40
19. 18.19	124.0390	C_20_H_38_	Neophytadiene	Diterpenoid	454,350.71
20. 17.21	256.2401	C_16_H_32_O_2_	n-Hexadecanoic acid	Fatty acid	1632,949.33
21. 17.21	154.1226	C_9_H_19_NO	Nonanamide	Amide	223,305.67
22. 22.39	340.2390	C_23_H_32_O_2_	Phenol, 2,2′-methylenebis[6-(1,1-dimethylethyl)-4-methyl-	Diterpenoid	40,545.33
23. 12.23	206.1636	C_14_H_22_O	Phenol, 2,5-bis(1,1-dimethylethyl)-	Sesquiterpenoid	29,976.67
24. 18.16	278.1512	C_20_H_30_O_4_	Phthalic acid, heptyl pentyl ester	Ester	4299,525.00
25. 17.11	223.0966	C_21_H_25_NO_3_	Phthalic acid, monoamide, N-ethyl-N-(3-methylphenyl)-, isobutyl ester	Ester	197,285.67
26. 19.62	278.2964	C_20_H_40_O	Phytol	Diterpenoid	658,427.67
27. 29.10	331.0378	C_29_H_48_O	Stigmasta-5,24(28)-dien-3-ol, (3β,24Z)-	Steroid	57,701.00
28. 28.61	412.3719	C_29_H_48_O	Stigmasterol	Steroid	701,624.67
29. 21.80	130.8943	C_10_H_16_O_2_	Tetrahydrofuran-2-one, 3-[2-pentenyl]-4-methyl-	Fatty acid ester	65,973.00
30. 19.56	85.0282	C_17_H_30_O_3_	Tetrahydropyran Z-10-dodecenoate	Ester	18,107.50
31. 17.68	218.9267	C_14_H_28_O_2_	Tridecanoic acid, methyl ester	Methyl ester	119,263.00
32. 27.60	431.3843	C_31_H_52_O_3_	α-Tocopheryl acetate	Triterpenoid	70,903.00
33. 29.01	414.3875	C_29_H_50_O	β-Sitosterol	Triterpenoid	1380,935.00

**Table 11:** Compounds identified in leaf acetone extracts of *Trichilia dregeana*.

RT (min)	Observed Ion *m*/*z*	MF	Name	MC	FC
1. 20.94	272.2504	C_20_H_32_	(R,1E,5E,9E)-1,5,9-Trimethyl-12-(prop-1-en-2-yl)cyclotetradeca-1,5,9-triene	Diterpenoid	661,369.50
2. 29.36	263.7976	C_13_H_20_N_2_SSi	1,2-Benzisothiazol-3-amine, TBDMS derivative	Sugar	24,657.75
3. 5.25	109.1014	C_8_H_14_	1,6-Heptadiene, 2-methyl-	Alkadiene	241,431.50
4. 18.65	277.2445	C_20_H_34_O	1H-Naphtho[2,1-b]pyran, 3-ethenyldodecahydro-3,4a,7,7,10a-pentamethyl-, [3R-(3α,4aβ,6aα,10aβ,10bα)]-	Triterpenoid	254,957.50
5. 21.38	218.9118	C_15_H_26_O	1-Naphthalenemethanol, 1,4,4a,5,6,7,8,8a-octahydro-2,5,5,8a-tetramethyl-	Sesquiterpenoid	910,101.00
6. 22.82	292.1670	C_17_H_24_O_4_	2-Hydroxy-4-methoxy-7-methyl-7,8,9,10,11,12,13,14-octahydro-6-oxabenzocyclododecen-5-one	Gingerdione	53,596.00
7. 16.78	180.1858	C_13_H_26_O	2-Undecanone, 6,10-dimethyl-	Fatty aldehyde	498,290.33
8. 21.78	263.8585	C_21_H_40_O_2_	4,8,12,16-Tetramethylheptadecan-4-olide	Beta-diketone	288,631.33
9. 29.60	281.9882	C_17_H_30_OSi	4-tert-Octylphenol, TMS derivative	Alkylbenzene	32,218.33
10. 21.69	270.2347	C_20_H_30_	Bicyclo[3.1.1]hept-2-ene, 2,2′-(1,2-ethanediyl)bis[6,6-dimethyl-	Diterpenoid	205,660.50
11. 23.33	218.8484	C_24_H_38_O_4_	Bis(2-ethylhexyl) phthalate	Ester	105,403.33
12. 21.69	289.2480	C_26_H_40_O_2_	Butyl 4,7,10,13,16,19-docosahexaenoate	Fatty acid	286,373.50
13. 20.88	263.9540	C_20_H_40_O_2_	Butyric acid, hexadecyl ester	Fatty acid	180,132.50
14. 28.14	226.1588	C_6_H_18_O_3_Si_3_	Cyclotrisiloxane, hexamethyl-	Organosilicon	21,468.33
15. 18.15	278.1513	C_16_H_22_O_4_	Dibutyl phthalate	Ester	5113,869.00
16. 15.29	87.0440	C_13_H_26_O_2_	Dodecanoic acid, 2-methyl-	Ester	161,611.50
17. 18.47	263.8584	C_20_H_42_	Eicosane	Alkane	491,217.33
18. 28.36	417.0340	C_30_H_50_O_2_	Ergost-5-en-3-ol, acetate, (3β,24R)-	Triterpenoid	303,607.00
19. 20.35	218.8367	C_27_H_56_	Heptacosane	Alkane	179,363.50
20. 13.54	130.8832	C_16_H_34_	Hexadecane	Alkane	578,509.80
21. 20.96	263.8346	C_21_H_44_O	Hexadecyl pentyl ether	Ether	415,977.00
22. 2.58	32.0175	H_4_N_2_	Hydrazine	Non-metal compound	195,016.33
23. 21.06	272.2508	C_20_H_32_	Kaur-15-ene	Diterpenoid	464,961.67
24. 2.89	32.0474	CH_4_O	Methyl Alcohol	Alcohol	3486,357.33
25. 16.70	218.8848	C_20_H_38_	Neophytadiene	Diterpenoid	115,963.50
26. 20.27	130.9004	C_9_H_19_NO	Nonanamide	Amide	132,986.33
27. 18.37	272.2506	C_20_H_32_	Phenanthrene, 7-ethenyl-1,2,3,4,4a,4b,5,6,7,9,10,10a-dodecahydro-1,1,4a,7-tetramethyl-, [4aS-(4aα,4bβ,7β,10aβ)]-	Diterpenoid	728,659.00
28. 17.09	223.0964	C_22_H_23_NO_4_	Phthalic acid, 4-cyanophenyl heptyl ester	Ester	230,352.00
29. 19.58	278.2973	C_20_H_40_O	Phytol	Diterpenoid	287,838.33
30. 28.57	412.3716	C_29_H_48_O	Stigmasterol	Steroid	308,190.00
31. 3.08	70.0412	C_2_H_4_OS	Thioacetic acid	Alkylthiol	687,016.50
32. 17.67	227.2003	C_14_H_28_O_2_	Tridecanoic acid, methyl ester	Fatty acid ester	219,513.33
33. 18.43	130.9689	C_4_BrF_9_	Tris(trifluoromethyl) bromomethane		19,641.50
34. 27.58	430.3812	C_29_H_50_O_2_	Vitamin E	Resorcinol	173,224.00
35. 21.06	218.8441	C_15_H_24_O	α-Santalol	7-hydroxycoumarin	82,416.00
36. 27.58	432.3888	C_31_H_52_O_3_	α-Tocopheryl acetate	Triterpenoid	186,539.25
37. 28.99	414.3867	C_29_H_50_O	β-Sitosterol	Triterpenoid	1328,446.00
38. 29.98	412.3719	C_29_H_48_O	γ-Sitostenone	Steroid	519,588.00

**Table 12:** Compounds identified in leaf ethanol extracts of *Trichilia dregeana*.

RT (min)	Observed Ion *m*/*z*	MF	Name	MC	FC
1. 13.97	218.9516	C_15_H_24_O	(1R,3E,7E,11R)-1,5,5,8-Tetramethyl-12-oxabicyclo[9.1.0]dodeca-3,7-diene	Epoxide	100,323.50
2. 20.96	275.2370	C_20_H_34_O	(E)-3-Methyl-5-((1R,4aR,8aR)-5,5,8a-trimethyl-2-methylenedecahydronaphthalen-1-yl)pent-2-en-1-ol	Diterpenoid	545,931.00
3. 22.71	118.9170	C_12_H_10_Cl_2_O_4_	1,2-Benzenediol, o-dichloroacetyl-o′-cyclopropanecarbonyl-		9,766.00
4. 30.62	266.9226	C_13_H_20_N_2_SSi	1,2-Benzisothiazol-3-amine, TBDMS derivative	Sugar	30,994.67
5. 26.17	280.9730	C_20_H_34_O	1,6,10,14-Hexadecatetraen-3-ol, 3,7,11,15-tetramethyl-, (E,E)-	Diterpenoid	320,433.80
6. 5.25	96.0564	C_8_H_14_	1,6-Heptadiene, 2-methyl-	Alkadiene	241,432.00
7. 18.67	276.2407	C_20_H_34_O	1H-Naphtho[2,1-b]pyran, 3-ethenyldodecahydro-3,4a,7,7,10a-pentamethyl-, [3R-(3α,4aβ,6aα,10aβ,10bα)]-	Triterpenoid	370,766.67
8. 23.14	218.8896	C_15_H_26_O	2,6,10-Dodecatrien-1-ol, 3,7,11-trimethyl-	Sesquiterpenoid	136,124.67
9. 12.49	218.9198	C_15_H_24_O	2,6,10-Dodecatrienal, 3,7,11-trimethyl-, (E,E)-	Sesquiterpenoid	141,636.50
10. 22.84	292.1672	C_17_H_24_O_4_	2-Hydroxy-4-methoxy-7-methyl-7,8,9,10,11,12,13,14-octahydro-6-oxabenzocyclododecen-5-one	Gingerdione	45,244.00
11. 16.80	179.1786	C_13_H_26_O	2-Undecanone, 6,10-dimethyl-	Fatty aldehyde	393,929.33
12. 20.91	263.8061	C_14_H_28_O_3_	3-Hydroxymyristic acid	Fatty acid	161,204.00
13. 21.21	274.2300	C_19_H_30_O	4,14-Dimethyl-11-isopropyltricyclo[7.5.0.0(10,14)]tetradec-4-en-8-one	Androgen	101,356.00
14. 21.80	263.9405	C_21_H_40_O_2_	4,8,12,16-Tetramethylheptadecan-4-olide	Beta-diketone	269,254.50
15. 11.17	130.8733	C_7_H_12_O	4-Hepten-2-one, (E)-	Organooxygen compound	136,106.00
16. 21.88	263.8605	C_18_H_35_NO	9-Octadecenamide, (Z)-	Fatty amide	708,408.50
17. 21.71	289.2489	C_26_H_40_O_2_	Butyl 4,7,10,13,16,19-docosahexaenoate	Fatty acid	318,610.00
18. 12.29	220.1824	C_15_H_24_O	Butylated Hydroxytoluene	Phenylpropane	27,910.67
19. 2.88	41.0132	CH_6_N_4_O	Carbohydrazide	Carbohydrazide	151,106.00
20. 23.54	257.2272	C_12_H_25_NO	Dodecanamide	Fatty amide	55,399.50
21. 17.69	227.2003	C_13_H_26_O_2_	Dodecanoic acid, methyl ester	Methyl ester	127,277.00
22. 2.62	31.0644	C_2_H_4_Cl_2_O	Ethanol, 2,2-dichloro-	Alcohol	12,654.00
23. 21.41	218.8306	C_15_H_26_O	Humulane-1,6-dien-3-ol	Sesquiterpenoid	1117,231.00
24. 11.43	130.9770	C_15_H_24_	Humulene	Sesquiterpenoid	33,834.00
25. 2.86	32.0543	H_4_N_2_	Hydrazine	Non-metal compound	29,779.50
26. 21.08	272.2510	C_20_H_32_	Kaur-15-ene	Diterpenoid	494,925.33
27. 2.65	31.9949	CH_4_O	Methyl Alcohol	Alcohol	1822,640.42
28. 15.82	171.1382	C_10_H_20_O_2_	n-Decanoic acid	Fatty acid	115,234.67
29. 16.72	137.1327	C_20_H_38_	Neophytadiene	Diterpenoid	139,113.50
30. 18.22	256.2398	C_16_H_32_O_2_	n-Hexadecanoic acid	Fatty acid	1293,680.33
31. 18.39	272.2504	C_20_H_32_	Phenanthrene, 7-ethenyl-1,2,3,4,4a,4b,5,6,7,9,10,10a-dodecahydro-1,1,4a,7-tetramethyl-, [4aS-(4aα,4bβ,7β,10aβ)]-	Diterpenoid	807,281.67
32. 22.39	340.2411	C_23_H_32_O_2_	Phenol, 2,2′-methylenebis[6-(1,1-dimethylethyl)-4-methyl-	Diterpenoid	67,053.50
33. 12.22	206.1663	C_14_H_22_O	Phenol, 2,5-bis(1,1-dimethylethyl)-	Sesquiterpenoid	41,210.00
34. 15.89	218.9111	C_3_F_9_P	Phosphine, tris(trifluoromethyl)-	Organofluorine	14,308.25
35. 18.17	278.1507	C_20_H_30_O_4_	Phthalic acid, heptyl pentyl ester	Ester	3770,875.00
36. 17.11	223.0962	C_21_H_25_NO_3_	Phthalic acid, monoamide, N-ethyl-N-(3-methylphenyl)-, isobutyl ester	Ester	244,482.50
37. 19.61	137.1326	C_20_H_40_O	Phytol	Diterpenoid	223,292.75
38. 28.34	380.3446	C_29_H_48_O	Stigmasta-5,24(28)-dien-3-ol, (3β,24Z)-	Steroid	234,342.50
39. 25.42	218.9552	C_30_H_50_	Supraene	Triterpenoid	565,315.33
40. 27.60	431.3852	C_31_H_52_O_3_	α-Tocopheryl acetate	Triterpenoid	264,498.80
41. 29.01	414.3875	C_29_H_50_O	β-Sitosterol	Triterpenoid	1371,309.00
42. 30.00	412.3724	C_29_H_48_O	γ-Sitostenone	Steroid	540,348.67
43. 27.05	416.3666	C_28_H_48_O_2_	γ-Tocopherol	Steroid	121,387.50

**Table 13:** Compounds identified in leaf acetone extracts of *Turraea floribunda*.

RT (min)	Observed Ion *m*/*z*	MF	Name	MC	FC
1. 15.00	220.1822	C_15_H_24_O	((4aS,8S,8aR)-8-Isopropyl-5-methyl-3,4,4a,7,8,8a-hexahydronaphthalen-2-yl)methanol	Sesquiterpenoid	171,920.00
2. 19.57	201.1638	C_15_H_24_	(1R,4S,5S)-1,8-Dimethyl-4-(prop-1-en-2-yl)spiro[4.5]dec-7-ene	Hydrocarbon	192,589.50
3. 26.14	263.8413	C_20_H_32_	(E,E,E)-3,7,11,15-Tetramethylhexadeca-1,3,6,10,14-pentaene	Diterpenoid	480,221.00
4. 21.84	273.2215	C_20_H_32_	1,3,6,10-Cyclotetradecatetraene, 3,7,11-trimethyl-14-(1-methylethyl)-, [S-(E,Z,E,E)]-	Diterpenoid	484,433.00
5. 20.78	201.1639	C_15_H_22_	1,3,7,11-Cyclotetradecatetraene, 2-methyl-		902,135.00
6. 18.80	263.7967	C_20_H_34_O	1,6,10,14-Hexadecatetraen-3-ol, 3,7,11,15-tetramethyl-, (E,E)-	Diterpenoid	650,540.67
7. 13.56	202.1714	C_15_H_26_	1H-3a,7-Methanoazulene, octahydro-1,4,9,9-tetramethyl-	Sesquiterpenoid	128,891.33
8. 8.68	142.0775	C_11_H_10_	1H-Indene, 1-ethylidene-	Hydrocarbon	28,659.00
9. 12.71	161.1324	C_11_H_16_O_2_	2(4H)-Benzofuranone, 5,6,7,7a-tetrahydro-4,4,7a-trimethyl-, (R)-	Benzofuran	135,928.67
10. 26.14	280.9475	C_22_H_36_O_2_	2,6,10,14-Hexadecatetraen-1-ol, 3,7,11,15-tetramethyl-, acetate, (E,E,E)-	Fatty alcohol	176,010.50
11. 15.19	210.1614	C_13_H_22_O_2_	2-Cyclohexen-1-one, 4-(3-hydroxybutyl)-3,5,5-trimethyl-	Apocarotenoid	82,345.33
12. 16.79	263.8863	C_18_H_36_O	2-Pentadecanone, 6,10,14-trimethyl-	Ketone	1520,735.33
13. 16.97	263.7692	C_20_H_40_O	3,7,11,15-Tetramethyl-2-hexadecen-1-ol	Diterpenoid	19,984.50
14. 25.67	218.8406	C_15_H_23_N	3-Cyano-3-octyl-1,4-cyclohexadiene		98,440.00
15. 5.24	105.0696	C_8_H_14_	4-Methyl-1,5-Heptadiene	Alkene	238,594.00
16. 12.47	221.1901	C_15_H_24_O	6,10-Dodecadien-1-yn-3-ol, 3,7,11-trimethyl-	Fatty alcohol	132,884.67
17. 20.45	263.8287	C_21_H_36_O_4_	9,12,15-Octadecatrienoic acid, 2,3-dihydroxypropyl ester, (Z,Z,Z)-	Lineolic acid	24,402.00
18. 21.75	288.2451	C_32_H_54_O_2_	9,19-Cyclolanostan-3-ol, acetate, (3β)-	Cycloartanol	185,601.00
19. 3.21	43.0106	C_2_H_6_N_2_O	Acetic acid, hydrazide	N-nitroso compound	9,648.50
20. 3.40	130.9333	C_2_H_4_O_3_	Acetic acid, hydroxy-	Hydroxy acid	16,192.00
21. 8.01	136.0518	C_8_H_8_O_2_	Benzeneacetic acid	Benzene	823,393.50
22. 20.33	218.8824	C_11_H_16_FNO_3_	Benzeneethanamine, 2-fluoro-β,3,4-trihydroxy-N-isopropyl-	Organofluorine compound	461,586.67
23. 21.65	274.2253	C_20_H_34_O_2_	Butyl 6,9,12-hexadecatrienoate	No records	120,874.00
24. 20.40	263.7774	C_20_H_30_O_2_	cis-5,8,11,14,17-Eicosapentaenoic acid	Fatty acid	67,970.33
25. 14.77	218.7973	C_15_H_24_O	cis-Z-α-Bisabolene epoxide	Sesquiterpenoid	163,987.00
26. 29.61	224.8923	C_6_H_18_O_3_Si_3_	Cyclotrisiloxane, hexamethyl-	Organosilicon	37,777.00
27. 6.10	130.9737	C_10_H_11_N_3_O_2_	dl-7-Azatryptophan	L-alpha-amino acid	5,636.50
28. 27.58	430.3826	C_29_H_50_O_2_	dl-α-Tocopherol	Resorcinol	308,112.50
29. 18.43	218.9230	C_20_H_42_	Eicosane	Alkane	773,942.50
30. 28.37	401.3748	C_30_H_50_O_2_	Ergost-5-en-3-ol, acetate, (3β,24R)-	Triterpenoid	400,755.50
31. 16.60	134.1088	C_12_H_18_	Geijerene	Monoterpenoid	506,492.50
32. 4.79	68.9660	C_3_H_8_O_3_	Glycerin	Sugar alcohol	1247,390.50
33. 20.35	263.9279	C_27_H_56_	Heptacosane	Alkane	459,191.00
34. 13.54	154.1719	C_16_H_34_	Hexadecane	Alkane	887,579.50
35. 2.58	32.0644	H_4_N_2_	Hydrazine	Non-metal compound	16,395.00
36. 21.65	218.9095	C_15_H_24_O	Isoaromadendrene epoxide	Sesquiterpenoid	87,763.50
37. 17.91	278.2969	C_20_H_40_O	Isophytol	Diterpenoid	581,608.33
38. 21.36	263.8432	C_22_H_34_O_2_	Methyl 6,9,12,15,18-heneicosapentaenoate	Methyl ester	222,155.00
39. 19.47	263.8150	C_18_H_30_O_2_	Methyl 8,11,14-heptadecatrienoate	Methyl ester	143,481.00
40. 2.63	32.0319	CH_4_O	Methyl Alcohol	Alcohol	2542,812.29
41. 16.70	218.9458	C_20_H_38_	Neophytadiene	Diterpenoid	455,672.53
42. 17.92	218.9160	C_18_H_36_O	Octadecanal	Fatty aldehyde	7,428.50
43. 22.38	340.2405	C_23_H_32_O_2_	Phenol, 2,2′-methylenebis[6-(1,1-dimethylethyl)-4-methyl-	Diterpenoid	98,778.00
44. 12.20	206.1665	C_14_H_22_O	Phenol, 2,6-bis(1,1-dimethylethyl)-	Sesquiterpenoid	26,409.00
45. 17.09	263.7937	C_22_H_23_NO_4_	Phthalic acid, 4-cyanophenyl heptyl ester	Ester	863,842.00
46. 23.33	279.1599	C_33_H_56_O_4_	Phthalic acid, heptadecyl 2-propylpentyl ester	Ester	192,600.50
47. 18.14	279.1561	C_20_H_30_O_4_	Phthalic acid, heptyl pentyl ester	Ester	7570,367.00
48. 2.96	31.4619	H_4_Si	Silane	Non-metal compound	14,625.00
49. 29.96	412.3726	C_29_H_48_O	Stigmast-4-en-3-one	Steroid	315,755.67
50. 25.39	231.2112	C_30_H_50_	Supraene	Triterpenoid	784,372.00
51. 15.80	218.9575	C_14_H_28_O_2_	Tetradecanoic acid	Fatty acid	201,837.50
52. 7.28	86.0223	C_4_H_6_S	Thiophene, 2,3-dihydro-	Dihydrothiophene	156,903.67
53. 13.54	218.8948	C_13_H_28_	Tridecane	Alkane	208,627.00
54. 17.65	228.2039	C_14_H_28_O_2_	Tridecanoic acid, methyl ester	Methyl ester	395,572.00
55. 12.99	157.1222	C_11_H_22_O_2_	Undecanoic acid	Methyl ester	51,909.00
56. 12.87	157.1011	C_15_H_20_	α-Calacorene	Sesquiterpenoid	30,917.33
57. 29.63	431.3858	C_31_H_52_O_3_	α-Tocopheryl acetate	Triterpenoid	199,285.00
58. 21.51	218.7617	C_15_H_24_O	β-Santalol	Sesquiterpenoid	80,032.50
59. 28.98	414.3873	C_29_H_50_O	β-Sitosterol	Triterpenoid	833,435.33

**Table 14:** Compounds identified in leaf ethanol extracts of *Turraea floribunda*.

RT (min)	Observed Ion *m*/*z*	MF.	Name	MC	FC
1. 28.27	218.8544	C_13_H_20_N_2_SSi	1,2-Benzisothiazol-3-amine, TBDMS derivative	Sugar	21,941.25
2. 16.77	130.9077	C_16_H_30_O_2_	2,15-Hexadecanedione	Fatty acid	8,739.00
3. 2.79	42.9883	C_2_H_5_ClO	2-Chloroethanol	Chloroethanol	17,013.50
4. 17.66	218.9181	C_9_H_16_BrNO	2-Piperidinone, N-[4-bromo-n-butyl]-	Delta-lactam	212,874.00
5. 16.78	218.8178	C_13_H_26_O	2-Undecanone, 6,10-dimethyl-	Fatty aldehyde	331,058.75
6. 30.93	250.8218	C_28_H_46_O_2_	4,4′-bi-4H-pyran, 2,2′,6,6′-tetrakis(1,1-dimethylethyl)-4,4′-dimethyl-		13,508.50
7. 27.65	206.9513	C_24_H_36_O_2_Si_2_	4-Methyl-2,4-bis(p-hydroxyphenyl)pent-1-ene, 2TMS derivative	Bisphenol A	25,492.75
8. 28.52	207.8478	C_17_H_30_OSi	4-tert-Octylphenol, TMS derivative	Alkylbenzene	23,331.67
9. 6.56	144.0417	C_6_H_8_O_4_	4H-Pyran-4-one, 2,3-dihydro-3,5-dihydroxy-6-methyl-	Fatty acid	123,337.50
10. 28.19	263.7777	C_9_H_27_AsO_3_Si_3_	Arsenous acid, tris(trimethylsilyl) ester	Trialkylheterosilane	17,467.00
11. 21.85	218.9263	C_11_H_16_FNO_3_	Benzeneethanamine, 2-fluoro-β,3,4-trihydroxy-N-isopropyl-	Organofluorine compound	197,067.50
12. 28.20	208.8548	C_6_H_18_O_3_Si_3_	Cyclotrisiloxane, hexamethyl-	Organosilicon	10,533.67
13. 18.93	130.8948	C_10_H_11_N_3_O_2_	dl-7-Azatryptophan	L-alpha-amino acid	13,803.00
14. 2.87	32.0230	H_4_N_2_	Hydrazine	Non-metal compound	29,112.33
15. 2.89	33.0078	H_3_NO	Hydroxylamine	Amine	19,835.67
16. 17.92	218.9493	C_20_H_40_O	Isophytol	Diterpenoid	126,936.00
17. 2.59	32.0438	CH_4_O	Methyl Alcohol	Alcohol	3945,937.00
18. 15.78	185.1534	C_10_H_20_O_2_	n-Decanoic acid	Fatty acid	56,292.00
19. 18.17	256.2398	C_16_H_32_O_2_	n-Hexadecanoic acid	Fatty acid	1236,320.33
20. 8.69	142.0773	C_11_H_10_	Naphthalene, 2-methyl-	Naphthalene	16,237.00
21. 16.71	137.1323	C_20_H_38_	Neophytadiene	Diterpenoid	212,701.60
22. 20.27	118.9582	C_9_H_19_NO	Nonanamide	Amide	135,588.33
23. 18.14	278.1516	C_20_H_30_O_4_	Phthalic acid, heptyl pentyl ester	Ester	4220,463.00
24. 19.59	278.2971	C_20_H_40_O	Phytol	Diterpenoid	332,925.75
25. 28.57	412.3722	C_29_H_48_O	Stigmasterol	Steroid	263,092.00
26. 19.68	218.8909	C_14_H_28_O_2_	Tridecanoic acid, methyl ester	Methyl ester	162,963.00
27. 28.28	218.8983	C_18_H_45_AsO_3_Si_3_	Tris(tert-butyldimethylsilyloxy)arsane	Ester	23,852.50
28. 17.66	199.1695	C_12_H_24_O_2_	Undecanoic acid, methyl ester	Methyl ester	230,369.50
29. 28.97	417.0367	C_31_H_52_O_2_	β-Sitosterol acetate	Triterpenoid	330,908.00

**Table 15:** Compounds identified in leaf acetone extracts of *Turraea obtusifolia*.

RT (min)	Observed Ion *m*/*z*	MF	Name	MC	FC
1. 15.00	220.1821	C_15_H_24_O	(1R,2R,4S,6S,7S,8S)-8-Isopropyl-1-methyl-3-methylenetricyclo[4.4.0.02,7]decan-4-ol	Sesquiterpenoid	126,668.00
2. 15.10	263.8326	C_18_H_26_O	1,3-Bis-(2-cyclopropyl,2-methylcyclopropyl)-but-2-en-1-one	2-benzopyran	92,518.50
3. 14.33	218.9465	C_15_H_24_O	10,10-Dimethyl-2,6-dimethylenebicyclo[7.2.0]undecan-5β-ol		219,693.33
4. 8.86	130.9685	C_10_H_16_O	2,4-Decadienal	Aldehyde	75,217.75
5. 16.78	179.1793	C_13_H_26_O	2-Undecanone, 6,10-dimethyl-	Fatty aldehyde	255,603.50
6. 11.98	202.1713	C_15_H_22_	3,5,11-Eudesmatriene	Sesquiterpenoid	56,152.67
7. 21.79	263.8648	C_21_H_40_O_2_	4,8,12,16-Tetramethylheptadecan-4-olide	Beta-diketone	410,582.00
8. 30.47	281.8219	C_17_H_30_OSi	4-tert-Octylphenol, TMS derivative	Alkylbenzene	27,817.50
9. 14.78	218.8204	C_15_H_24_O	6,10-Dodecadien-1-yn-3-ol, 3,7,11-trimethyl-	Fatty alcohol	206,334.00
10. 15.85	218.1667	C_15_H_22_O	7-Isopropenyl-1,4a-dimethyl-4,4a,5,6,7,8-hexahydro-3H-naphthalen-2-one		186,966.33
11. 20.00	263.9515	C_20_H_34_O_2_	8,11,14-Eicosatrienoic acid, (Z,Z,Z)-	Fatty acid	13,480.00
12. 19.94	280.2397	C_18_H_32_O_2_	9,12-Octadecadienoic acid (Z,Z)-	Fatty acid	44,571.00
13. 29.97	422.3937	C_32_H_52_O_2_	9,19-Cycloergost-24(28)-en-3-ol, 4,14-dimethyl-, acetate, (3β,4α,5α)-	Triterpenoid	119,424.50
14. 16.95	263.8819	C_20_H_30_O_5_	Andrographolide	Butyrolactone	182,421.67
15. 21.37	204.1875	C_15_H_24_	Azulene, 1,2,3,5,6,7,8,8a-octahydro-1,4-dimethyl-7-(1-methylethenyl)-, [1S-(1α,7α,8aβ)]-	Sesquiterpenoid	235,288.00
16. 8.67	142.0774	C_11_H_10_	Benzocycloheptatriene	Benzenoid	17,979.50
17. 27.85	218.8211	C_6_H_18_O_3_Si_3_	Cyclotrisiloxane, hexamethyl-	Organosilicon	19,618.33
18. 10.01	130.9654	C_10_H_11_N_3_O_2_	dl-7-Azatryptophan	L-alpha-amino acid	12,483.25
19. 13.94	218.8738	C_22_H_32_O_2_	Doconexent	Fatty acid	40,765.00
20. 18.43	263.7630	C_20_H_42_	Eicosane	Alkane	644,244.67
21. 11.70	202.1715	C_15_H_22_	Eudesma-2,4,11-triene	Sesquiterpenoid	186,595.60
22. 20.31	263.9065	C_16_H_33_NO	Hexadecanamide	Fatty amide	235,968.00
23. 20.35	218.7788	C_16_H_34_	Hexadecane	Alkane	444,080.00
24. 2.59	32.0455	H_4_N_2_	Hydrazine	Non-metal compound	27,402.00
25. 17.91	263.7523	C_20_H_40_O	Isophytol	Diterpenoid	180,211.33
26. 14.93	220.1819	C_15_H_24_O	Ledene oxide-(II)	Sesquiterpenoid	165,643.67
27. 2.86	32.0231	CH_4_O	Methyl Alcohol	Alcohol	3308,314.78
28. 16.70	218.9564	C_20_H_38_	Neophytadiene	Diterpenoid	239,419.44
29. 18.21	256.2402	C_16_H_32_O_2_	n-Hexadecanoic acid	Fatty acid	1312,119.00
30. 16.22	218.7640	C_15_H_32_	Pentadecane	Alkane	703,400.00
31. 6.40	130.9444	C_6_H_12_O_3_	Pentanoic acid, 2-hydroxy-3-methyl-	Fatty acid	374,290.67
32. 17.09	263.7686	C_21_H_23_NO_6_	Phthalic acid, heptyl 4-nitrophenyl ester	Ester	403,612.00
33. 18.14	279.1556	C_20_H_30_O_4_	Phthalic acid, heptyl pentyl ester	Ester	4790,918.75
34. 19.59	279.2999	C_20_H_40_O	Phytol	Diterpenoid	423,228.75
35. 28.57	412.3719	C_29_H_48_O	Stigmasterol	Steroid	467,141.50
36. 15.79	185.1536	C_14_H_28_O_2_	Tetradecanoic acid	Fatty acid	105,645.50
37. 3.07	70.0287	C_2_H_4_OS	Thioacetic acid	Alkylthiol	27,616.00
38. 17.66	227.2008	C_14_H_28_O_2_	Tridecanoic acid, methyl ester	Fatty acid ester	449,472.00
39. 19.68	218.8289	C_12_H_24_O_2_	Undecanoic acid, methyl ester	Methyl ester	180,225.75
40. 27.58	430.3823	C_29_H_50_O_2_	Vitamin E	Resorcinol	262,653.50
41. 29.64	432.3904	C_31_H_52_O_3_	α-Tocopheryl acetate	Triterpenoid	295,293.67
42. 25.67	420.3571	C_29_H_50_O_4_	α-Tocospiro A	Steroid	141,624.33
43. 28.98	414.3868	C_29_H_50_O	β-Sitosterol	Triterpenoid	999,333.33

**Table 16:** Compounds identified in leaf ethanol extracts of *Turraea obtusifolia*.

RT (min)	Observed Ion m/z	MF	Name	MC	FC
1. 13.98	220.1814	C_15_H_24_O	(1R,3E,7E,11R)-1,5,5,8-Tetramethyl-12-oxabicyclo[9.1.0]dodeca-3,7-diene	Epoxide	360,103.67
2. 20.96	275.2379	C_20_H_34_O	(E)-3-Methyl-5-((1R,4aR,8aR)-5,5,8a-trimethyl-2-methylenedecahydronaphthalen-1-yl)pent-2-en-1-ol	Diterpenoid	115,266.00
3. 21.30	263.7396	C_17_H_32_O	(R)-(-)-14-Methyl-8-hexadecyn-1-ol	Fatty alcohol	73,991.50
4. 14.35	218.8756	C_15_H_24_O	10,10-Dimethyl-2,6-dimethylenebicyclo[7.2.0]undecan-5β-ol		385,059.67
5. 17.93	218.8876	C_20_H_40_O	1-Hexadecen-3-ol, 3,5,11,15-tetramethyl-	Diterpenoid	315,859.50
6. 12.74	177.0783	C_11_H_16_O_2_	2(4H)-Benzofuranone, 5,6,7,7a-tetrahydro-4,4,7a-trimethyl-	Benzofuran	93,776.33
7. 12.38	155.0938	C_8_H_13_NO_2_	2-Hydroxy-1-(1′-pyrrolidiyl)-1-buten-3-one		99,138.00
8. 16.80	179.1793	C_13_H_26_O	2-Undecanone, 6,10-dimethyl-	Fatty aldehyde	295,979.67
9. 11.99	202.1717	C_15_H_22_	3,5,11-Eudesmatriene	Sesquiterpenoid	84,655.00
10. 16.73	178.9410	C_20_H_40_O	3,7,11,15-Tetramethyl-2-hexadecen-1-ol	Diterpenoid	23,305.00
11. 21.81	263.8249	C_21_H_40_O_2_	4,8,12,16-Tetramethylheptadecan-4-olide	Beta-diketone	348,560.00
12. 6.57	144.0418	C_6_H_8_O_4_	4H-Pyran-4-one, 2,3-dihydro-3,5-dihydroxy-6-methyl-	Fatty acid	842,545.00
13. 28.80	280.8152	C_17_H_30_OSi	4-tert-Octylphenol, TMS derivative	Alkylbenzene	31,758.67
14. 28.39	400.3725	C_28_H_48_O	5-Cholestene-3-ol, 24-methyl-	Steroid	548,943.50
15. 13.48	218.8472	C_15_H_24_O	6,10-Dodecadien-1-yn-3-ol, 3,7,11-trimethyl-	Fatty alcohol	153,763.25
16. 15.88	218.1668	C_15_H_22_O	7-Isopropenyl-1,4a-dimethyl-4,4a,5,6,7,8-hexahydro-3H-naphthalen-2-one		307,202.33
17. 20.97	272.2511	C_18_H_30_O_2_	9,12,15-Octadecatrienoic acid, (Z,Z,Z)-	Methyl ester	923,805.50
18. 19.49	218.9427	C_19_H_32_O_2_	9,12,15-Octadecatrienoic acid, methyl ester, (Z,Z,Z)-	Methyl ester	152,852.00
19. 19.88	263.7534	C_18_H_32_O_2_	9,12-Octadecadienoic acid (Z,Z)-	Fatty acid	14,775.50
20. 2.95	32.0576	C_2_H_4_O_3_	Acetic acid, hydroxy-	Hydroxy acid	8,158.50
21. 13.85	218.8142	C_15_H_24_O	Bergamotol, Z-α-trans-	Monoterpenoid	48,437.33
22. 13.60	218.8457	C_15_H_24_O	Caryophyllene oxide	Sesquiterpenoid	487,571.00
23. 14.61	218.8863	C_15_H_24_O	cis-Z-α-Bisabolene epoxide	Sesquiterpenoid	177,591.67
24. 31.24	218.9091	C_6_H_18_O_3_Si_3_	Cyclotrisiloxane, hexamethyl-	Organosilicon	22,405.75
25. 29.66	430.3832	C_29_H_50_O_2_	dl-α-Tocopherol	Resorcinol	429,663.00
26. 17.67	213.1853	C_13_H_26_O_2_	Dodecanoic acid, methyl ester	Methyl ester	353,733.33
27. 11.72	202.1716	C_15_H_22_	Eudesma-2,4,11-triene	Sesquiterpenoid	160,053.60
28. 13.27	130.9365	C_12_H_9_Cl_3_O_4_	Fumaric acid, ethyl 3,4,5-trichlorophenyl ester		53,322.00
29. 3.46	58.0088	C_4_H_8_O_4_	Glycolaldehyde dimer	Pentose	11,108.50
30. 14.96	220.1826	C_15_H_24_O	Ledene oxide-(II)	Sesquiterpenoid	335,514.67
31. 14.61	263.9254	C_19_H_32_O_3_	Methyl 2-hydroxy-octadeca-9,12,15-trienoate	Fatty acid	365,830.00
32. 2.97	32.0260	CH_4_O	Methyl Alcohol	Alcohol	3178,893.00
33. 16.72	221.1896	C_20_H_38_	Neophytadiene	Diterpenoid	260,778.86
34. 18.26	256.1901	C_16_H_32_O_2_	n-Hexadecanoic acid	Fatty acid	5461,473.67
35. 20.34	156.0941	C_9_H_19_NO	Nonanamide	Amide	370,402.80
36. 12.24	206.1660	C_14_H_22_O	Phenol, 2,6-bis(1,1-dimethylethyl)-	Sesquiterpenoid	60,234.00
37. 18.16	278.1520	C_20_H_30_O_4_	Phthalic acid, heptyl pentyl ester	Ester	7217,745.00
38. 19.61	278.2960	C_20_H_40_O	Phytol	Diterpenoid	507,674.67
39. 7.29	130.9626	C_7_H_13_NO_2_	Pyrrolidin-1-propionic acid	Proline	87,679.50
40. 7.28	133.1012	C_6_H_12_ClN	Pyrrolidine, 1-(2-chloroethyl)-	Haloalkyl	88,402.00
41. 28.60	412.3717	C_29_H_48_O	Stigmasterol	Steroid	585,313.00
42. 20.16	227.2011	C_14_H_28_O_2_	Tetradecanoic acid	Fatty acid	200,040.00
43. 14.81	219.9886	C_15_H_24_O	trans-Z-α-Bisabolene epoxide	Sesquiterpenoid	242,292.25
44. 27.60	430.3830	C_29_H_50_O_2_	Vitamin E	Resorcinol	309,983.50
45. 25.67	421.3605	C_29_H_50_O_4_	α-Tocospiro A	Steroid	220,431.00
46. 29.01	414.3884	C_29_H_50_O	β-Sitosterol	Triterpenoid	1141,657.00

**Table 17:** Scale used to assign different classes of percentage repulsion values [75].

Class	Percentage Repulsion
0	>0.01 to <0.1
I	0.1–20
II	20.1–40
III	40.1–60
IV	60.1–80
V	80.1–100

## Data Availability

Not applicable.
